# Recognition of a translocation motif in the regulator HpaA from *Xanthomonas euvesicatoria* is controlled by the type III secretion chaperone HpaB

**DOI:** 10.3389/fpls.2022.955776

**Published:** 2022-07-28

**Authors:** Sabine Drehkopf, Christian Otten, Daniela Büttner

**Affiliations:** Institute for Biology, Department of Genetics, Martin Luther University Halle-Wittenberg, Halle (Saale), Saxony-Anhalt, Germany

**Keywords:** type III secretion, plant pathogen, chaperone, translocation signal, sorting platform, gatekeeper

## Abstract

The Gram-negative plant-pathogenic bacterium *Xanthomonas euvesicatoria* is the causal agent of bacterial spot disease in pepper and tomato plants. Pathogenicity of *X. euvesicatoria* depends on a type III secretion (*T3S*) system which translocates effector proteins into plant cells and is associated with an extracellular pilus and a translocon in the plant plasma membrane. Effector protein translocation is activated by the cytoplasmic *T3S* chaperone HpaB which presumably targets effectors to the *T3S* system. We previously reported that HpaB is controlled by the translocated regulator HpaA which binds to and inactivates HpaB during the assembly of the *T3S* system. In the present study, we show that translocation of HpaA depends on the *T3S* substrate specificity switch protein HpaC and likely occurs after pilus and translocon assembly. Translocation of HpaA requires the presence of a translocation motif (TrM) in the N-terminal region. The TrM consists of an arginine-and proline-rich amino acid sequence and is also essential for the *in vivo* function of HpaA. Mutation of the TrM allowed the translocation of HpaA in *hpaB* mutant strains but not in the wild-type strain, suggesting that the recognition of the TrM depends on HpaB. Strikingly, the contribution of HpaB to the TrM-dependent translocation of HpaA was independent of the presence of the C-terminal HpaB-binding site in HpaA. We propose that HpaB generates a recognition site for the TrM at the *T3S* system and thus restricts the access to the secretion channel to effector proteins. Possible docking sites for HpaA at the *T3S* system were identified by *in vivo* and *in vitro* interaction studies and include the ATPase HrcN and components of the predicted cytoplasmic sorting platform of the *T3S* system. Notably, the TrM interfered with the efficient interaction of HpaA with several *T3S* system components, suggesting that it prevents premature binding of HpaA. Taken together, our data highlight a yet unknown contribution of the TrM and HpaB to substrate recognition and suggest that the TrM increases the binding specificity between HpaA and *T3S* system components.

## Introduction

The Gram-negative plant-pathogenic bacterium *Xanthomonas euvesicatoria* (also known as *Xanthomonas campestris* pv. *vesicatoria*) is the causal agent of bacterial spot disease on pepper and tomato plants and causes significant yield losses due to necrotic lesions and defoliation (Potnis et al., 2015). Pathogenicity of *X. euvesicatoria* depends on a type III secretion (*T3S*) system which is conserved in many Gram-negative plant-and animal-pathogenic bacteria and translocates bacterial effector proteins (T3Es, type III effectors) into eukaryotic cells (Büttner and Bonas, 2002; An et al., 2020; Timilsina et al., 2020). *T3S* systems span both bacterial membranes and are associated with an extracellular pilus-like structure which serves as a transport channel for secreted proteins to eukaryotic cells (Büttner, 2012; Deng et al., 2017; Hajra et al., 2021). Translocation of T3Es is mediated by a bacterial *T3S* translocon in the eukaryotic plasma membrane (Büttner, 2012; Ji and Dong, 2015; Dey et al., 2019). T3Es interfere with various cellular pathways to the benefit of the pathogen, thus allowing bacterial multiplication and the formation of disease symptoms in susceptible plants. In resistant plants, however, individual T3Es are directly or indirectly recognized by the plant immune system (Büttner, 2016; Khan et al., 2018; Schreiber et al., 2021). Corresponding plant resistance proteins subsequently activate defence responses which often lead to the induction of a hypersensitive response (HR), a rapid local cell death at the infection site that restricts bacterial multiplication (Khan et al., 2016; Ngou et al., 2022).

The *T3S* system from *X. euvesicatoria* is encoded by a chromosomal *hrp* (HR and pathogenicity) gene cluster which contains 25 genes organized in eight operons (Büttner and Bonas, 2002). Eleven gene products are conserved in animal-and/or plant-pathogenic bacteria and presumably constitute the core elements of the secretion apparatus. They are, therefore, referred to as Hrc for Hrp conserved (Hueck, 1998). Homologous proteins from animal-pathogenic bacteria are designated Sct (secretion and cellular translocation) followed by a letter corresponding to the nomenclature of *T3S* system components from *Yersinia* spp. (Hueck, 1998; Büttner, 2012; Wagner and Diepold, 2020). In several animal-pathogenic bacteria, the contribution of Sct proteins to the formation of membrane-associated and cytoplasmic core components of the *T3S* system has been intensively studied (Deng et al., 2017; Wagner et al., 2018; Lara-Tejero and Galan, 2019; Milne-Davies et al., 2021). Structural and electron microscopy studies revealed that the *T3S* system consists of multimeric rings in the inner membrane (IM) and outer membrane (OM) which are connected by a periplasmic inner rod (Worrall et al., 2016; Hu et al., 2017, 2018, 2019; Torres-Vargas et al., 2019; Lunelli et al., 2020). The IM rings surround the export apparatus which is presumably situated above the IM in the periplasm and is assembled by SctR, SctS, SctT, SctU and SctV proteins (Dietsche et al., 2016; Zilkenat et al., 2016; Kuhlen et al., 2018; Johnson et al., 2019). The IM proteins SctU and SctV contain large cytoplasmic domains which are likely involved in substrate docking (Büttner, 2012; Sal-Man et al., 2012; Portaliou et al., 2017; Wagner et al., 2018; Xing et al., 2018; Milne-Davies et al., 2021). The IM rings also associate with the cytoplasmic sorting platform which is a dynamic structure and consists of the ATPase complex, six spoke-like structures formed by SctL dimers and six pods assembled by members of the SctQ protein family (Hu et al., 2015, 2017; Lara-Tejero, 2019; Berger et al., 2021).

The sorting platform presumably engages *T3S* substrates, however, the mechanisms underlying substrate recognition and substrate docking are not yet understood (Akeda and Galan, 2005; Spaeth et al., 2009; Lara-Tejero et al., 2011). Studies in animal-pathogenic bacteria suggest that substrate recruitment by the sorting platform involves *T3S* chaperones which bind to and stabilize secreted proteins and facilitate the entry of their interaction partners into the secretion channel (Büttner, 2012; Deng et al., 2017; Wagner et al., 2018). Substrates of *T3S* systems include extracellular components of the *T3S* system such as pilus and translocon proteins as well as T3Es. Given the architecture of the *T3S* system, secretion of pilus and translocon proteins presumably precedes translocation of T3Es, suggesting a hierarchy in *T3S* (Deng et al., 2005; Winnen et al., 2008; Lara-Tejero et al., 2011; Portaliou et al., 2017). Recognition of *T3S* substrates by the *T3S* system depends on the presence of a secretion signal which is often located in the N-terminal region and is not conserved on the amino acid level (Sory et al., 1995; Rüssmann et al., 2002; Löwer and Schneider, 2009; Samudrala et al., 2009; Wang et al., 2013). In addition to the *T3S* signal, T3Es contain a translocation signal for delivery across the eukaryotic cell membrane (Schesser et al., 1996; Cheng et al., 1997; Boyd et al., 2000; Stebbins and Galan, 2001; Schechter et al., 2004).

In *X. euvesicatoria*, *T3S* substrate specificity is regulated by the control protein HpaC which promotes secretion of translocon and effector proteins and inhibits the secretion of the predicted inner rod protein HrpB2 (Lorenz et al., 2008b; Lorenz and Büttner, 2011; Schulz and Büttner, 2011; Hartmann et al., 2012). The HpaC-mediated substrate specificity switch is likely controlled by a conformational change in the cytoplasmic domain of the export apparatus component HrcU, HrcU_C_. A point mutation in HrcU_C_, which presumably mimics the predicted conformational change, restores *T3S* in the absence of HpaC, suggesting that the contribution of HpaC to *T3S* is mediated by HrcU_C_ (Lorenz and Büttner, 2011). Given that HrcU_C_ interacts with the N-terminal region of HrpB2, which contains the *T3S* signal, a conformational change in HrcU_C_ might alter substrate recognition by the *T3S* system (Lorenz and Büttner, 2011; Scheibner et al., 2016).

It is still unknown how T3Es are recognized by the *T3S* system after the HpaC-mediated *T3S* substrate specificity switch. *T3S* system-mediated delivery of T3Es depends on translocation signals which have been identified in the N-terminal 100–200 amino acids (Büttner et al., 2006; Schulze et al., 2012; Scheibner et al., 2017, 2018). Furthermore, many T3Es contain an N-terminal characteristic amino acid pattern, termed translocation motif (TrM), which harbours arginine-and proline-rich sequences (Escolar et al., 2001; Prochaska et al., 2018). The TrM was previously identified as a membrane targeting signal in the T3E XopB and was shown to contribute to the translocation of XopB, AvrBs1 and AvrBsT (Escolar et al., 2001; Prochaska et al., 2018). Interestingly, mutation of the TrM in XopB abolished membrane localization and facilitated translocation of XopB in the absence of the *T3S* chaperone HpaB (Prochaska et al., 2018). It was, therefore, proposed that HpaB releases XopB from the membrane in order to facilitate its export by the *T3S* system. HpaB was previously shown to interact with and to promote the translocation of different T3Es (Büttner et al., 2004; Schulze et al., 2012; Lonjon et al., 2017; Scheibner et al., 2018; Gan et al., 2021). Notably, however, HpaB also interacts with several cytoplasmic components of the *T3S* system, suggesting an association with the sorting platform (Büttner et al., 2004; Lorenz and Büttner, 2009). HpaB-effector complexes are presumably dissociated by the ATPase HrcN, which might thus facilitate the entry of effectors into the inner transport channel (Lorenz and Büttner, 2009; Gan et al., 2021).

We previously suggested that the contribution of HpaB to T3E delivery is controlled by HpaA which binds to HpaB and acts as a secreted regulator (Lorenz et al., 2008a). HpaA contributes to *T3S* and pathogenicity and is itself translocated into the plant cell where it localizes to the nucleus (Huguet et al., 1998; Lorenz et al., 2008a). Nuclear localization of HpaA, however, is likely dispensable for its virulence function because the insertion of nuclear exclusion signals, which abolished nuclear localization of HpaA, did not affect the *in vivo* activity of HpaA (Lorenz et al., 2008a). We, therefore, speculate that the virulence function of HpaA is attributed to its role as regulator of HpaB. Previous interaction studies showed that the C-terminal region of HpaA contains a binding site for HpaB and is essential for protein function (Lorenz et al., 2008a). The interaction between HpaA and HpaB likely prevents an inhibitory effect of HpaB on *T3S* during the assembly of the *T3S* system. This hypothesis is supported by the previous finding that deletion of *hpaB* in a *hpaA* mutant restored *T3S* of extracellular components of the *T3S* system (Lorenz et al., 2008a). Furthermore, N-terminal deletion derivatives of HpaA, which are secretion-deficient but interact with HpaB, still promote secretion of translocon proteins but not of T3Es (Lorenz et al., 2008a). Thus, secretion and translocation of HpaA after assembly of the *T3S* system is presumably required to liberate HpaB and to activate HpaB-dependent T3E delivery. The timing of HpaA secretion and translocation is, therefore, a key event for the control of T3E delivery in *X. euvesicatoria.*

In the present study, we analysed the translocation signal and TrM of HpaA and its contribution to the docking of HpaA to *T3S* system components. We show that the TrM is essential for HpaA translocation and *in vivo* function. Interaction studies revealed that the TrM interferes with the efficient binding of HpaA to *T3S* system components, suggesting that it controls the binding specificity of HpaA. Interestingly, mutation of the TrM allowed HpaA translocation in the absence but not the presence of HpaB. We, therefore, propose that HpaB is involved in the recognition of the TrM by the *T3S* system.

## Materials and methods

### Bacterial strains and growth conditions

Bacterial strains and plasmids used in this study are listed in Supplementary Table S1. *Escherichia coli* strains were grown at 37°C in lysogeny broth (LB) medium, and *X. euvesicatoria* was grown at 30°C in nutrient-yeast extract-glycerol (NYG) medium or minimal medium A (MA; 12 mM K_2_HPO_4_, 6.6 mM KH_2_PO_4_, 1.52 mM (NH_4_)_2_SO_4_, 0.34 mM Na-citrate*2H_2_O, 1 mM MgSO_4_; pH 7.0) supplemented with sucrose (10 mM) and casamino acids (0.3%). Antibiotics were added to the media at the following final concentrations: ampicillin, 100 μg/ml; kanamycin, 25 μg/ml; rifampicin, 100 μg/ml; spectinomycin, 100 μg/ml, gentamycin, 15 μg/ml; streptomycin, 25 μg/ml and nalidixic acid, 15 μg/ml. Plasmids were introduced into *E. coli* by transformation and into *X. euvesicatoria* by electroporation or triparental conjugation, using pRK2013 as helper plasmid.

### Plant material and plant infections

For infection studies, *X. euvesicatoria* strains were resuspended in 1 mM MgCl_2_ at an optical density (OD_600nm_) of 0.1 [corresponding to 1 × 10^8^ colony-forming units (CFU) ml^−1^] if not stated otherwise and infiltrated into leaves of the near-isogenic pepper cultivars Early Cal Wonder (ECW), ECW-10R and ECW-30R using a needleless syringe. Infected plants were incubated in growth chambers for 16 h of light at 28°C and 8 h of darkness at 22°C. Disease symptoms and the hypersensitive response (HR) were documented over a period of one to 9 days post inoculation (dpi). For a better visualization of the HR, infected leaves were bleached in 70% ethanol. Results of the infection experiments were reproduced at least two times.

### Generation of expression constructs

For the generation of *hpaA* expression constructs, *hpaA* and derivatives were amplified by PCR from *X. euvesicatoria* strain 85–10 and cloned into the Golden Gate-compatible expression vector pBRM using the type IIs restriction enzyme *Bsa*I in a single restriction/ligation reaction (Engler et al., 2008). Vector pBRM allows the expression of genes under control of a *lac* promoter in frame with a 3 x c-Myc epitope-encoding sequence. Similarly, *hpaA* and derivatives thereof were cloned into the *Bsa*I sites of plasmid pBR356, which contains the *avrBs3∆2* reporter gene (Scheibner et al., 2016). To introduce the mutations in the TrM, the 5′ and 3′ regions of *hpaA* were amplified as separate fragments using primers that annealed back-to-back at the TrM-encoding sequence and contained the mutations. For bacterial adenylate cyclase-based two-hybrid (BACTH) assays, *hpaA* and derivatives thereof were cloned into the Golden Gate-compatible vectors pUT18_GG_, pUT18C_GG_, pKT25_GG_ and pKNT25_GG_. Additionally, we amplified *hpaB*, *hpaC* and *hrcU_C_* by PCR from *X. euvesicatoria* strain 85–10, subcloned the corresponding amplicons into pICH41021 as blunt-end fragments after *Sma*I digest and ligation, and cloned the resulting inserts into the *Bsa*I sites of vectors pUT18_GG_, pUT18C_GG_, pKT25_GG_ and pKNT25_GG_ using Golden Gate cloning. Primers used in this study are listed in Supplementary Table S2.

### Analysis of *in vitro T3S*

*In vitro* *T3S* assays with *X. euvesicatoria* strains were performed as described previously (Rossier et al., 1999). For this, bacteria were grown overnight in minimal medium A (MA) (pH 7.0) supplemented with sucrose (10 mM) and casamino acids (0.3%) and shifted to MA (pH 5.3; *T3S* permissive) containing 50 μg ml^−1^ bovine serum albumin (BSA) and 10 μM thiamine at an optical density at 600 nm (OD_600_) of 0.2 for 4 h. Cultures were incubated on a rotary shaker at 30°C, and bacterial cells and secreted proteins from 6 ml-cultures were separated by filtration. Proteins in 4 ml of the culture supernatants were precipitated with trichloroacetic acid and resuspended in 20 μl of Laemmli buffer. Total cell extracts and culture supernatants were analysed by SDS-PAGE and immunoblotting, using antibodies directed against the c-Myc epitope as well as against HrpF and HrcJ, respectively (Rossier et al., 1999; Büttner et al., 2002). Horseradish peroxidase-labelled anti-mouse and anti-rabbit antibodies were used as secondary antibodies. Binding of antibodies was visualized using a chemiluminescence imager (Vilber Fusion FX Edge). Experiments were performed three times with similar results.

### Glutathione S-transferase (GST) pull-down assays

For GST pull-down assays, *E. coli* BL21 (DE3) cells expressing the potential interaction partners under control of the *lac* promoter were grown in LB medium until an OD_600_ of 0.6–0.8 and gene expression was induced in the presence of IPTG (isopropyl-*β*-D-thiogalactopyranoside; 2 mM final concentration) for 2 h at 37°C. Bacterial cells were harvested by centrifugation, broken with a French press and cell debris were removed by an additional centrifugation step. GST and GST fusion proteins present in the cell lysates were immobilized on a glutathione sepharose matrix according to the manufacturer’s instructions (GE Healthcare). After washing of the matrix, immobilized GST and GST fusion proteins were incubated with bacterial lysates containing the predicted interaction partner for 2 h at 4°C on an overhead shaker. Unbound proteins were removed by washing the sepharose matrix and bound proteins were eluted with Laemmli buffer. Cell lysates and eluted proteins were analysed by SDS-PAGE and immunoblotting using antibodies directed against the c-Myc epitope and GST, respectively. Horseradish peroxidase-labelled anti-mouse and anti-goat antibodies were used as secondary antibodies. Binding of antibodies was visualized using a chemiluminescence imager (Vilber Fusion FX Edge). Experiments were performed three times with similar results.

### Fluorescence microscopy analyses

*X. euvesicatoria* strains were grown overnight in MA medium (pH 7.0) supplemented with sucrose (10 mM) and casamino acids (0.3%). Cells were resuspended in MA medium (pH 5.3) supplemented with BSA and thiamine at an OD_600_ of 0.15 and the cultures were incubated on a tube rotator at 30°C for at least 1 h. Bacteria were transferred to a microscopy slide on top of a pad of 1.5% agarose dissolved in MA medium (pH 5.3) as described (Hausner et al., 2019). Fluorescence was inspected using a confocal laser scanning microscope (Leica STELLARIS 8) with a 60 x magnification objective and 5 x digital magnification. The fluorophores were excited with WLL (white light laser) according to their wave lengths (sfGFP excitation at 485 nm; mCherry excitation at 587 nm). Fluorescence signals were detected with HyD S detectors with adjusted detection windows corresponding to emission of the fluorophores (sfGFP emission at 510 nm; mCherry emission at 610 nm). Pictures were taken from approximately 100 cells of three transconjugants for each strain and were processed with the Leica LIGTHNING detection concept to reveal or sharpen fine structures and details. Protein synthesis was analysed by SDS-PAGE and immunoblotting using GFP- (Roche), mCherry- (Abcam), HrpB1-and HrcC-specific antibodies (Wengelnik et al., 1996; Rossier et al., 2000).

### BACTH assays

For BACTH assays, expression constructs encoding T25 and T18 fusion proteins were transferred to the BTH101 *E. coli* reporter strain and transformants were selected on LB medium containing kanamycin and gentamycin. At least three colonies per strain were used to inoculate LB overnight cultures with appropriate antibiotics, which were incubated at 30°C on a rotary shaker. Two microlitres of the overnight cultures were spotted on LB plates containing appropriate antibiotics, X-gal (5-bromo-4-chloro-3-indolyl-β-D-galactopyranosid; 40 μg/ml) and 2 mM IPTG. Plates were photographed after five to 6 days of incubation at room temperature. The results of all BACTH assays were reproduced at least two times with new transformants; one representative colony is shown. To detect T18 and T25 fusions, corresponding expression constructs were transferred to JM109 *E. coli* cells and bacterial cultures were induced with IPTG at an OD_600_ of 0.6–0.8 and incubated on a rotary shaker for 2 h at 37°C. Bacterial cells were collected by centrifugation, resuspended in Laemmli buffer and analysed by immunoblotting, using a FLAG epitope-specific antibody.

### Coimmunoprecipitation experiments in *X. euvesicatoria*

For coimmunoprecipitation studies, bacteria were grown over night in 50 ml MA medium (pH 5.3) supplemented with sucrose (10 mM) and casamino acids (0.3%). Similar amounts of bacterial cells adjusted to the same optical density were harvested by centrifugation, resuspended in 1 ml PBS supplemented with proteinase inhibitors (PIC, proteinase inhibitor cocktail, Roche) and broken with a french press. To dissolve proteins, 0.1% Nonidet-P40 was added to the cell lysate. After 15 min of incubation on ice, cell debris were removed by centrifugation and the supernatant was incubated with 20 μl of protein G agarose beads (Roche) for 1 h at 4°C. The mixture was centrifuged for 90 s at 700 × *g* and the supernatants were incubated with 60 μl of protein G agarose in the presence or absence of 4 ng anti-c-Myc antibody over night at 4°C on an overhead shaker. The protein G agarose was washed three times with PBS buffer and resuspended in 40 μl of Laemmli buffer. Cell lysates and immunoprecipitated proteins were analysed by immunoblotting, using c-Myc epitope-and HrcN-specific antibodies.

### Generation of modular *hrp* gene cluster constructs for the analysis of HpaA-sfGFP

The modular *hrp* gene cluster constructs used for fluorescence microscopy were generated as described previously (Hausner et al., 2019) using the modular cloning system (Weber et al., 2011) based on the Golden Gate cloning method (Engler et al., 2009). For the generation of the *hpaA-2xLinker-sfgfp* module, *hpaA* was amplified with primers hpaA-NTM-MoClo-F and hpaA-NTM-MoClo-R and the amplicon was cloned into vector pAGM9121 using *Bp*iI and ligase, thus resulting in the level-2 construct pAGB1302. This module was subsequently cloned using *Bsa*I and ligase into vector pAGM1311 to create the level-1 construct pAGB1304, which finally allows the generation of translational fusions of *hpaA* with genes encoding C-terminal fusion partners. In the next step, *hpaA* (construct pAGB1304), a linker sequence (2 × AKLEGPAGL, construct pAGB1000) and *sfgfp* (construct pAGB997) were cloned using *Bpi*I and ligase into vector pICH41308, thus generating the level 0 construct pAGB1328, which contains *hpaA-2xLinker-sfgfp*. The insert of pAGB1328 (*hpaA-2xLinker-sfgfp*) was transferred together with the inserts of constructs pAGB249 (*PhrpD*) and pAGB232 (*Xcv term2*) into the destination vector pICH47742 using *Bsa*I and ligase and resulting in the level 1 construct pAGB1330 (*PhrpD hpaA-2xLinker-sfgfp*). The level 0 construct pAGB232 was generated by amplifying the terminator (*Xcv term2*) with primers termX2-euk-MoClo-F and termX2-euk-MoClo-R and cloning the PCR product into the vector pICH41276 using *Bpi*I and ligase. The insert of pAGB1330 (*PhrpD hpaA-2xLinker-sfgfp*) was then ligated with the inserts of pAGB157 (*xopA* + *hpaH*), pAGB160 (*hrpX*), pAGB163 (hrpG*), pICH54066 (dummy pos. 6) and pICH50881 (end linker) into the *Bpi*I sites of the level M vector pAGM8079, leading to the level M construct pAGB1332.

To introduce a *hpaA* deletion and *hpaA*/*hpaB* double deletion into the modular gene cluster, a *hpaA* deletion module was generated by PCR using primers hpaA-Del-MoClo-F and hrcD-Del-MoClo-R3. The resulting PCR fragment (contains *hrcD* and ∆*hpaA*) was cloned into vector pAGM9121 using *Bpi*I and ligase, resulting in the level-2 construct pAGB1323 (*hrcD*, ∆*hpaA*). For the generation of the *hpaB* deletion, the original module pAGB208 (*hrpE*, *hpaB*) was separated by amplifying *hrpE* with primers hpaB-Del-MoClo-I-F and hpaB-Del-MoClo-I-R and the fragment containing the deletion in *hpaB* with primers hpaB-Del-MoClo-II-F and hpaB-Del-MoClo-II-R. The PCR fragments were individually cloned using *Bpi*I and ligase into pAGM9121 to generate the level-2 constructs pAGB649 (*hrpE*) and pAGB650 (∆*hpaB*). For the introduction of a deletion in *hpaA*, the insert of pAGB1323 (*hrcD*, ∆*hpaA*) was ligated with the inserts of pAGB205 (*hrcS*), pAGB207 (*hrpD6*), pAGB208 (*hrpE*, *hpaB*) and pAGB209 (*hpaE*) using *Bsa*I and ligase into vector pAGM1311 to create the level-1 construct pAGB1324. For the *hpaA*/*hpaB* double deletion the same modules were inserted into vector pAGM1311, except for the pAGB208 construct, which was replaced by constructs pAGB649 (*hrpE*) and pAGB650 (∆*hpaB*) resulting in the level-1 construct pAGB1359. The inserts of the resulting constructs pAGB1324 (*hrcS* to *hpaE* region with a deletion in *hpaA*) and pAGB1359 (*hrcS* to *hpaE* region with a deletion in *hpaA* and *hpaB*) were individually assembled with the insert of construct pAGB210 (*PhrpC, hrcU* to *hrcR*) into the vector pICH41331 using *Bpi*I and ligase to generate the level 0 constructs pAGB1325 (*hrpC* to *hpaB* operons with a deletion in *hpaA*) and pAGB1360 (*hrpC* to *hpaB* operons with a deletion in *hpaA* and *hpaB*). In a following step, each of these inserts was transferred using *Bsa*I and ligase into the level 1 destination vector pICH47751, resulting in the level 1 constructs pAGB1326 (*hrpC to hpaB* operons with a deletion in *hpaA*) and pAGB1361 (*hrpC* to *hpaB* operons with a deletion in *hpaA* and *hpaB*). The inserts of pAGB1326 or pAGB1361 were subsequently cloned together with the inserts of pAGB154 (*hrpA* to *hrpB* operons), pAGB156 (*hrpF* operon), pICH54011 (dummy pos.1), pICH50900 (end linker) into the *Bpi*I sites of vector pAGM8031 to generate the level M constructs pAGB1327 (*hrp* gene cluster with a deletion in *hpaA*) and pAGB1362 (*hrp* gene cluster with a deletion in *hpaA* and *hpaB*).

Finally, the level M construct pAGB1332 (contains *xopA*, *hpaH*, *hrpX*, hrpG* and *hpaA-2xLinker-sfgfp*) was assembled with the end linker pICH79264 and the level M construct pAGB1327 (*hrp* gene cluster with a deletion in *hpaA*) or pAGB1362 (*hrp* gene cluster with a deletion in *hpaA* and *hpaB*) in the level P vector pICH75322 using *Bsa*I and ligase, thus resulting in the level P constructs pAGB1334 (contains *hpaA-2xLinker-sfgfp* and a deletion in *hpaA*) and pAGB1374 (contains *hpaA-2xLinker-sfgfp* and a deletion in *hpaA* and *hpaB*).

### Generation of modular *hrp* gene cluster constructs for the analysis of peripheral protein localization in bacterial cells

As control for peripheral protein localization in bacterial cells (periplasm or membrane-associated), a *hrcC-2xLinker-mCherry* module was generated. For this, *hrcC* was amplified with primers hrcC-NTM-MoClo-F and hrcC-NTM-MoClo-R and the resulting amplicon was cloned using *Bpi*I and ligase into vector pAGM9121 to generate the level-2 construct pAGB611. The insert of pAGB611 was subsequently transferred into vector pAGM1311 using *Bsa*I and ligase to create the level-1 construct pAGB474. This module allows the generation of translational fusions of *hrcC* to genes encoding potential C-terminal fusion partners. *mCherry* as potential fusion partner was amplified using primers mCherry-CTM-woL-F and mCherry-CTM-woL-R, and the resulting PCR fragment was cloned into vector pICH41021 using *Sma*I and ligase to generate the level-1 construct pAGB1048. The inserts of constructs pAGB474 (*hrcC*), pAGB1048 (*mCherry*) and pAGB1000 (encodes linker sequence 2 × AKLEGPAGL) were cloned using *Bpi*I and ligase into vector pICH41308, thus resulting in the level 0 construct pAGB1059. In the next step, the inserts of constructs pAGB1059 (*hrcC-2xLinker-mcherry*), pAGB232 (*Xcv term2*) and pAGB511 (*PhrpA*) were assembled in the destination vector pICH47781 using *Bsa*I and ligase, thus generating the level 1 construct pAGB1063. The native *hrpA* promoter (*PhrpA*) was amplified with primers PhrpA-MoClo-F and PhrpA-MoClo-R and the resulting PCR product was cloned as blunt-end fragment using *Sma*I and ligase into vector pICH41021 to generate the level-1 construct pAGB614. The insert of pAGB614 was subsequently inserted into the *Bpi*I sites of vector pICH41295 to generate the level 0 construct pAGB511 (*PhrpA*). The insert of pAGB1063 (*PhrpA hrcC-2xLinker-mcherry*) was assembled with the inserts of pAGB157 (*xopA* + *hpaH*), pAGB160 (*hrpX*), pAGB163 (hrpG*), pICH54022 (dummy pos. 2′) and pICH50881 (end linker) in vector pAGM8079 using *Bpi*I and ligase, resulting in the level M construct pAGB1067.

For the analysis of HrcC-mCherry localization, *hrcC* was deleted in the modular *hrp* gene cluster. For this, we generated a *hrcC* deletion module by PCR using the phosphorylated primers P-hrcC-Del-MoClo-F and P-hrcC-Del-MoClo-R and pAGB196 (*hrcC*) as template. Ligation of the PCR product resulted in the level-2 construct pAGB515. The insert of pAGB515 (∆*hrcC*) was then assembled with the inserts of constructs pAGB192 (*hrcL*), pAGB193 (*hrcN*), pAGB194 (*hrpB7*) and pAGB195 (*hrcT*) into vector pAGM1311 using *Bsa*I and ligase to create the level-1 construct pAGB676. The insert of construct pAGB676 (contains *hrcL* to *hrcT* and *hrcC* with a deletion) was subsequently assembled with the insert of pAGB197 (contains *hrpB1* to *hrpB4*) in the *Bpi*I sites of vector pICH41331, resulting in the level 0 construct pAGB680. The insert of pAGB680 (*hrpA* and *hrpB* operon with a *hrcC* deletion) was transferred into the destination vector pICH47811 using *Bsa*I and ligase, thus resulting in the level 1 construct pAGB683. To generate the level M construct containing the *hrp* gene cluster with a *hrcC* deletion, the inserts of constructs pAGB683 (*hrpA* to *hrpB* operons with a deletion in *hrcC*), pAGB155 (*hrpC*, *hrpD*, *hrpE* and *hpaB* operons), pAGB156 (*hrpF* operon), pICH54011 (dummy pos.1) and pICH50900 (end linker) were assembled in the *Bpi*I sites of vector pAGM8031, resulting in the level M construct pAGB739. In a final cloning step, the inserts of constructs pAGB739 (*hrp* gene cluster with a *hrcC* deletion), pAGB1067 (*hrcC-2xLinker-mcherry*) and pICH79264 (end linker) were cloned into the *Bsa*I sites of vector pICH75322, thus resulting in the level P construct pAGB1075.

## Results

### The N-terminal 70 amino acids of HpaA including the TrM are essential for translocation

**Figure 1:**
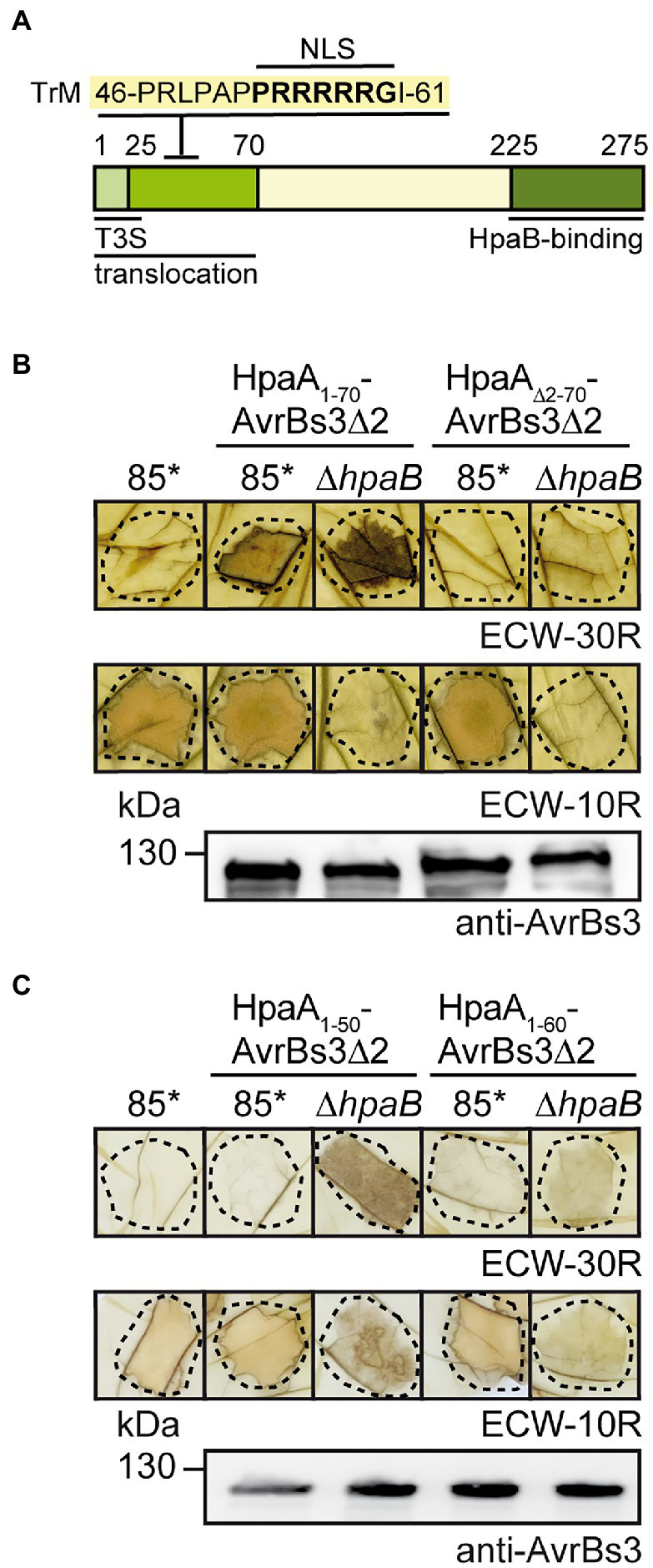
The N-terminal region is essential for HpaA translocation. **(A)** Schematic representation of functional regions in HpaA. Letters refer to the amino acid sequence of the N-terminal TrM, numbers indicate amino acid positions. The localization of the N-terminal *T3S* and translocation signal and the C-terminal HpaB-binding site is indicated (Lorenz et al., 2008a). TrM, translocation motif. **(B)** The N-terminal 70 amino acids are essential for HpaA translocation. *X. euvesicatoria* strains 85* and 85*∆*hpaB* with or without expression constructs encoding HpaA_1-70_-AvrBs3∆2 or HpaA_∆2-70_-AvrBs3∆2 as indicated were infiltrated into leaves of AvrBs3-responsive ECW-30R and AvrBs1-responsive ECW-10R pepper plants. Leaves were bleached in ethanol 2 dpi. Dashed lines indicate the infiltrated areas. For protein analysis, bacterial strains were grown in nutrient-yeast extract-glycerol (NYG) medium and equal amounts of protein extracts were analysed by immunoblotting using an AvrBs3-specific antiserum. **(C)** The N-terminal 50 amino acids of HpaA contain a minimal translocation signal. *X. euvesicatoria* strains 85* and 85*∆*hpaB* with or without expression constructs encoding HpaA_1-50_-AvrBs3∆2 or HpaA_1-60_-AvrBs3∆2 as indicated were infiltrated into leaves of AvrBs3-responsive ECW-30R and AvrBs1-responsive ECW-10R pepper plants. HR reactions and protein synthesis were documented as described in **(B)**.

We previously showed that the N-terminal 70 amino acids of HpaA are sufficient to target a reporter protein for translocation (Lorenz et al., 2008a). This region contains a characteristic amino acid pattern (46-PRLRPAPPRRRRRGI-61), designated TrM, with a Pro/Arg-rich sequence, which is also present in the N-terminal regions of T3Es from *X. euvesicatoria* (Figure 1A). In HpaA, the TrM overlaps with an N-terminal predicted nuclear localization (NLS1) motif (amino acids 54–60) which is one out of two putative NLS motifs and presumably involved in nuclear targeting of HpaA after translocation into the plant cells (Figure 1A; Huguet et al., 1998; Lorenz et al., 2008a).

To further study the translocation signal of HpaA, we generated fusion proteins between HpaA derivatives and the reporter protein AvrBs3∆2, which is an N-terminal deletion derivative of the TAL effector AvrBs3 and lacks the *T3S* export signal. AvrBs3∆2 is only translocated when fused to a functional translocation signal and, hence, induces the HR in AvrBs3-responsive pepper plants, which contain the *R* gene *Bs3* (Szurek et al., 2002; Noël et al., 2003). We first investigated whether the N-terminal 70 amino acids are essential for translocation of HpaA. For this, an AvrBs3∆2 reporter fusion containing HpaA deleted in amino acids 2–70 was analysed in *X. euvesicatoria* strain 85-10hrpG* (85*). Strain 85* contains a constitutively active version of the key regulator HrpG, which allows the analysis of *in vitro* *T3S* and the elicitation of accelerated plant reactions (Wengelnik et al., 1999). When bacteria were infiltrated into leaves of AvrBs3-responsive pepper plants, HpaA_1-70_-AvrBs3∆2 but not HpaA_∆2-70_-AvrBs3∆2 induced a visible HR, suggesting that the N-terminal 70 amino acids are sufficient and essential for translocation of HpaA (Figure 1B). Immunoblot analysis of bacterial cell extracts showed that HpaA_∆2-70_-AvrBs3∆2 was stably synthesized (Figure 1B).

We also generated expression constructs containing the N-terminal 50 (lacking the TrM) or 60 (including the Pro/Arg-rich sequence) amino acids of HpaA as fusion partners of AvrBs3∆2. When analysed in strain 85*, HpaA_1-50_-AvrBs3∆2 and HpaA_1-60_-AvrBs3∆2 were stably synthesized but did not elicit a visible HR in AvrBs3-responsive pepper plants (Figure 1C). This is in contrast to HpaA_1-70_-AvrBs3∆2, which is translocated by the wild-type strain (Lorenz et al., 2008a), suggesting that the N-terminal 50 and 60 amino acids of HpaA and thus the Pro/Arg-rich sequence were not sufficient to target the AvrBs3∆2 reporter for translocation. It can, however, not be excluded that additional amino acids present in HpaA_1-70_-AvrBs3∆2 are required for a correct exposure of the TrM on the protein surface and thus its recognition by the *T3S* system. Notably, when analysed in the *hpaB* deletion mutant strain 85*∆*hpaB*, HpaA_1-50_-AvrBs3∆2 induced the AvrBs3-specific HR, indicating that the N-terminal 50 amino acids of HpaA contain a minimal translocation signal which is recognized in the absence of HpaB but not in the wild-type strain (Figure 1C). Similar findings were previously observed for N-terminal regions of the effector proteins AvrBs3, XopE2 and XopJ (Scheibner et al., 2017, 2018). Interestingly, N-terminal minimal translocation signals do not include the TrM (Table 1).

**Table 1:** Contribution of TrM sequences in T3Es from *X. euvesicatoria* to *T3S* and translocation.

			T3S^3^		Translocation^4^		
Protein	TrM^1^	Signal^2^					References
			WT	∆*hpaB*	WT	∆*hpaB*	
XopB	SRLPTTRPPRRRSTSSG (46–63)	R/A TrM^-^P/A	+/− +/− +/−	++ ++ ++	+/− +/− +/−	++ ++ +	Prochaska et al., 2018
AvrBs3	GPLDGLPARRTMSRT (37–52)	1–30 1–40 1–50	+ + +	+ + +	– – –	+ + +	Scheibner et al., 2017
XopE2	PSLHGLVALGSSGTRR (43–59)	1–40 1–50	+ +	n.a. n.a.	– –	– +	Scheibner et al., 2018
XopJ	SGLPERVALKTKLLA (63–78)	1–50	+	n.a.	−	+	Scheibner et al., 2018
HpaA	PRLRPAPPRRRRRGI (46–61)	1–50 1–60 1–70 TrMmut ∆2–70	n.a. n.a. + + n.a.	n.a. n.a. n.a. n.a. n.a.	– – + – –	+ – + + –	This study

1 Amino acid sequences and the position of the TrM are given.

2 Protein regions, which were analysed as fusion partners of reporter proteins, are indicated. Numbers refer to N-terminal amino acids which were analysed as fusion partners of the reporter protein AvrBs3∆2. R/A, Arg residues of the TrM of XopB were substituted by Ala; P/A, Pro residues of the TrM were exchanged with Ala residues, TrM^−^, the TrM was replaced by alanine residues (Prochaska et al., 2018).

3 *In vitro* *T3S* was analysed in strains 85* (WT) and 85*∆*hpaB* (∆*hpaB*). Protein regions were analysed as fusion partners of the reporter protein AvrBs3∆2. +, wild-type secretion; +/−, reduced secretion; n.a., not analysed.

4 For the analysis of *in vivo* translocation, protein regions were analysed as fusion partners of the reporter protein AvrBs3∆2 in strains 85* (WT) and 85*∆*hpaB* (∆*hpaB*). +, translocation detectable in ECW-30R pepper plants; +/−, reduced translocation; −, no translocation detectable.

### The contribution of the TrM to translocation of HpaA depends on the *T3S* chaperone Hpab

**Figure 2:**
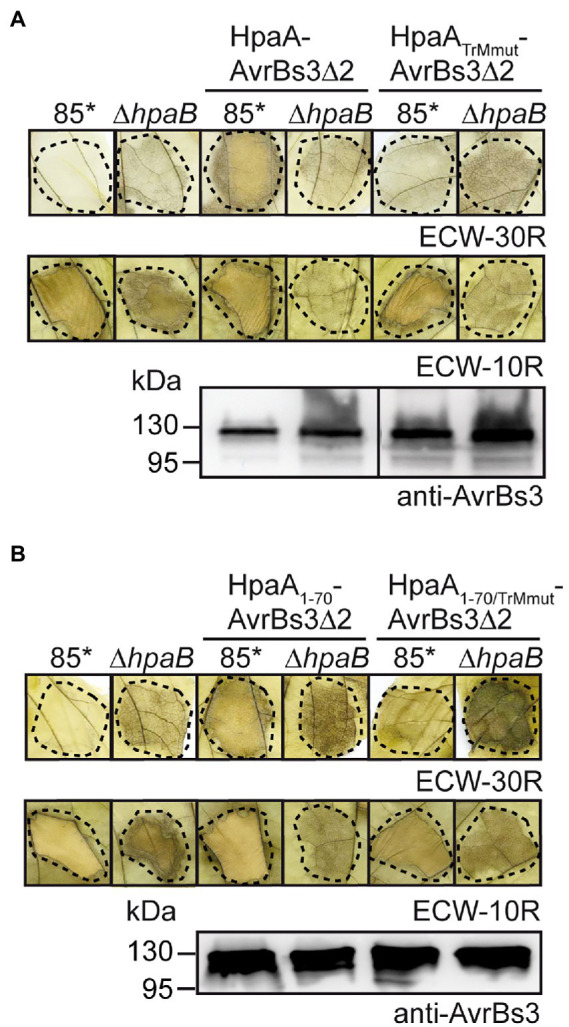
The N-terminal TrM determines HpaB-dependent translocation of HpaA. **(A)** The TrM is essential for HpaA translocation. *X. euvesicatoria* strains 85* and 85*∆*hpaB* with or without expression constructs encoding HpaA-AvrBs3∆2 or HpaA_TrMmut_-AvrBs3∆2 as indicated were infiltrated at a density of 4 × 10^8^  cfu ml^−1^ into leaves of AvrBs3-responsive ECW-30R and AvrBs1-responsive ECW-10R pepper plants. Leaves were bleached in ethanol 2 dpi. Dashed lines indicate the infiltrated areas. For protein analysis, bacteria were grown in NYG medium, and equal amounts of protein extracts were analysed by immunoblotting using an AvrBs3-specific antiserum. **(B)** Mutation of the TrM promotes translocation of HpaA in the absence of HpaB. *X. euvesicatoria* strains 85* and 85*∆*hpaB* with or without expression constructs encoding HpaA_1-70_-AvrBs3∆2 or HpaA_1-70/TrMmut_-AvrBs3∆2 as indicated were infiltrated into leaves of AvrBs3-responsive ECW-30R and AvrBs1-responsive ECW-10R pepper plants. HR reactions and protein synthesis were documented as described in **(A)**.

To further investigate the contribution of the TrM to HpaA translocation, we introduced point mutations leading to the exchange of amino acids 54-PRRRRRG-60 to 54-PAAAAAG-60 (hereafter referred to as TrMmut) in HpaA-AvrBs3∆2 and HpaA_1-70_-AvrBs3∆2. These mutations abolished the detectable translocation of both fusion proteins by strain 85* but did not interfere with protein stability as was shown by immunoblot analyses of bacterial cell extracts (Figures 2A,B). Interestingly, however, the TrM mutation promoted translocation of HpaA-AvrBs3∆2 fusions in strain 85*∆*hpaB* (Figures 2A,B). This is in agreement with the above observation that HpaA_1-50_-AvrBs3∆2 lacking the TrM is translocated by strain 85*∆*hpaB* but not by the wild-type strain (Figure 1C). Notably, the contribution of the TrM to HpaA translocation is independent of HpaB binding because the HpaB-binding site is located in the C-terminal 50 amino acids of HpaA (Lorenz et al., 2008a). Our findings are reminiscent of the previous observation that mutation of the TrM in the effector protein XopB interfered with XopB translocation in the wild-type strain but led to enhanced translocation of XopB reporter fusions in the absence of HpaB (Prochaska et al., 2018; Table 1). The authors postulated that the TrM mutation abolished the membrane localization of XopB and thus bypassed the need of XopB for the predicted HpaB-mediated release from the membrane (Prochaska et al., 2018). It is, however, unknown whether the TrM also serves as a membrane localization signal in other T3Es.

### HpaA does not localize to the bacterial membranes

To investigate whether HpaA localizes to the bacterial membranes as was suggested for XopB, we analysed a HpaA–sfGFP fusion protein in *X. euvesicatoria* by fluorescence microscopy. For this, we used a modular expression construct containing the *T3S* gene cluster, regulatory (hrpG*, *hrpX*) and accessory genes (the putative translocon gene *xopA* and the lytic transglycosylase gene *hpaH*), which allows the assembly and activation of the *T3S* system in *X. euvesicatoria* (Noël et al., 2002; Hausner et al., 2017, 2019). The modular *T3S* gene cluster was assembled from single modules containing promoters and genes using the Golden Gate-based modular cloning system as described previously (Hausner et al., 2019). For this, promoter and gene modules were assembled in different cloning steps by alternating the use of the type IIs restriction enzymes *Bsa*I and *Bpi*I. This led to level 0 constructs containing single or multiple operons, which were flanked by identical fusion sites. For further assembly, level 0 modules were transferred to different level 1 vectors which allowed subsequent positional cloning into a level M vector. Two level M modules corresponding to the *hrp* gene cluster and the regulatory and accessory genes, respectively, were finally assembled in a level P vector (Hausner et al., 2019).

**Figure 3:**
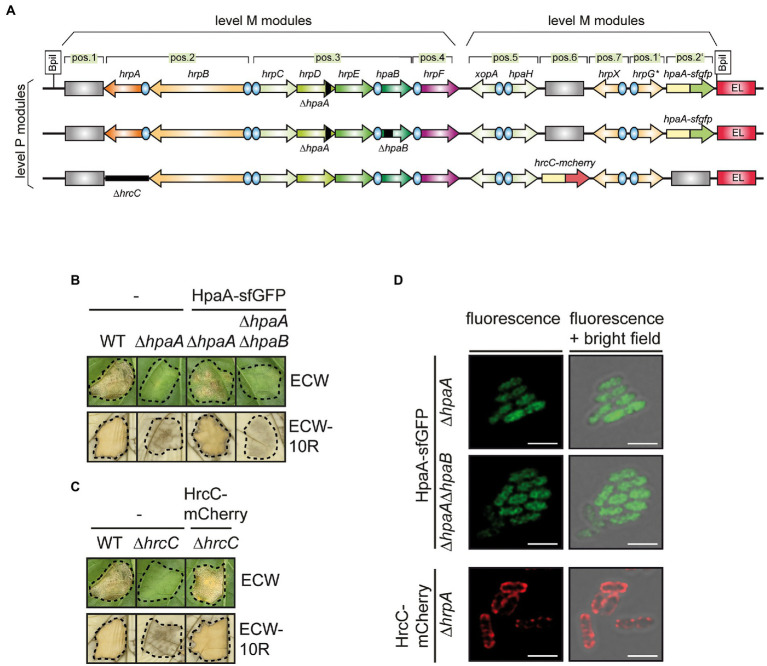
Localization studies with a HpaA-sfGFP fusion protein. **(A)** Schematic representation of modular *T3S* gene cluster constructs. Genes and operons are represented by arrows, promoters by blue circles. Grey rectangles represent dummy modules that were replaced by reporter fusions. Deletions are indicated with black rectangles. The names of single operons and genes is given above the arrows. The modular level P constructs resulted from the assembly of two level M modules. **(B)** Complementation studies with HpaA-sfGFP. Strain 85*∆*hrp* containing the wild-type modular *T3S* gene cluster (WT) or a derivative thereof with a deletion in *hpaA* (∆*hpaA*) and strain 85*∆*hrp_fsHAGX* containing modular *T3S* gene cluster constructs with deletions in *hpaA* or both *hpaA* and *hpaB* (∆*hpaAB*) and encoding HpaA-sfGFP in the flanking region of the *T3S* gene cluster as depicted in **(A)** were infiltrated into leaves of susceptible ECW and resistant ECW-10R pepper plants. Dashed lines indicate the infiltrated areas. Disease symptoms were photographed 8 dpi. For the better visualization of the HR, leaves were bleached in ethanol 2 dpi. HpaA-sfGFP was stably synthesized as is shown in Supplementary Figure S1. **(C)** HrcC-mCherry complements the *hrcC* mutant phenotype. Strain 85*∆*hrp* containing the wild-type modular *T3S* gene cluster (WT) or a derivative thereof with a deletion in *hrcC* (∆*hrcC*) and strain 85*∆*hrp_fsHAGX* containing the modular *T3S* gene cluster deleted in *hrcC* and encoding HrcC-mCherry in the flanking region of the *T3S* gene cluster as depicted in **(A)** were infiltrated into leaves of susceptible ECW and resistant ECW-10R pepper plants. Plant reactions were monitored as described in **(B)**. HrcC-mCherry was stably synthesized as is shown in Supplementary Figure S1. **(D)** HpaA-sfGFP is evenly distributed in bacterial cells. *X. euvesicatoria* strain 85*∆*hrp_fsHAGX* with plasmids containing the modular *T3S* gene cluster, accessory and regulatory genes with deletions in *hpaA*, *hpaA* and *hpaB* or *hrcC* and *hpaA-sfgfp* or *hrcC-mcherry* inserted into the flanking region of the modular *T3S* gene cluster as indicated was incubated under *T3S*-permissive conditions and analysed by fluorescence microscopy. One representative image for every strain is shown. The size bar corresponds to 2 μm. The pictures in the right panels result from an overlay of the signals from the fluorescent channel for GFP with the bright field images. For better visualisation of fine structures, the confocal pictures were processed with the LIGHTNING technology of Leica. Experiments were performed with different transconjugants for each strain three times with similar results. The results from one representative experiment are shown.

The modular design of the construct facilitates the rapid deletion and insertion of single gene and promoter modules and thus the analysis of reporter fusions. For the experiments with HpaA-sfGFP, the corresponding gene fusion was inserted into the flanking region of the modular *T3S* gene cluster which was deleted in the native *hpaA* gene (Figure 3A). The resulting construct was transferred to *X. euvesicatoria* strain 85*∆*hrp_ fsHAGX* which lacks the chromosomal *T3S* gene cluster and contains mutations in the native *hrpG, hrpX, xopA* and *hpaH* genes (Hausner et al., 2019). To analyse whether HpaA-sfGFP was functional, bacteria were infiltrated into leaves of resistant and susceptible pepper plants. As expected, the wild-type strain induced the HR and water-soaked lesions, respectively, whereas plant reactions were significantly reduced after infection with a strain containing the modular *T3S* gene cluster deleted in *hpaA* (Figure 3B). The wild-type phenotype was restored upon insertion of *hpaA-sfGFP* into the modular construct, suggesting that the sfGFP fusion partner did not significantly interfere with HpaA function (Figure 3B). Immunoblot analysis of bacterial cell extracts showed that HpaA-sfGFP was stably synthesized (Supplementary Figure S1).

For localization studies, bacteria were incubated under *T3S*-permissive conditions and analysed by fluorescence microscopy. The GFP fluorescence was evenly distributed in all cells (Figure 3D). A similar localization pattern was observed upon additional deletion of *hpaB*, suggesting that HpaB does not significantly affect the localization of HpaA (Figure 3D). As control for the localization studies, we generated an additional construct expressing the modular *T3S* gene cluster deleted in the native secretin gene *hrcC* and containing a *hrcC-mCherry* fusion in the flanking region (Figure 3A). mCherry is a red fluorescent protein suitable for the analysis of periplasmic proteins (Shaner et al., 2004; Meiresonne et al., 2017, 2019). The secretin HrcC contains a Sec signal for the transport across the IM and forms an oligomeric protein channel in the OM (Wengelnik et al., 1996; Silva et al., 2020). Infection and immunoblot experiments showed that HrcC-mCherry was stably synthesized and complemented the *hrcC* mutant phenotype *in planta* (Figure 3C and Supplementary Figure S1). When analysed by fluorescence microscopy, HrcC-mCherry localized to the periphery of the bacterial cells as expected and also formed fluorescent spots likely corresponding to assembled secretin channels in the OM (Figure 3D). The clear difference in the fluorescence signals of HrcC-mCherry and HpaA-sfGFP indicates that both proteins do not colocalize at the cell periphery. We, therefore, conclude that HpaA does not primarily localize to the bacterial membranes as was reported for XopB. The absence of detectable fluorescent foci for HpaA-sfGFP, which would indicate an association with assembled *T3S* systems, suggests that HpaA is not exclusively attached to the secretion apparatus but also present in the bacterial cytoplasm.

### The TrM is dispensable for secretion but essential for the *in vivo* function of HpaA

**Figure 4:**
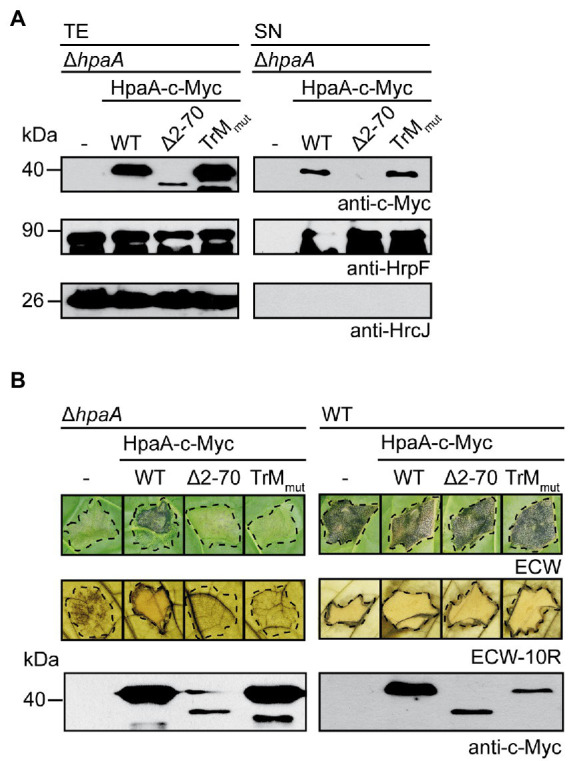
The TrM is essential for the *in vivo* function of HpaA. **(A)** Mutation of the TrM does not interfere with *T3S* of HpaA. *X. euvesicatoria* strain 85*∆*hpaA* (∆*hpaA*) without expression construct (−) or containing HpaA-c-Myc (WT), HpaA_∆2-70_-c-Myc (∆2–70) or HpaA_TrMmut_-c-Myc (TrMmut) as indicated was incubated under *T3S*-permissive conditions. Total cell extracts (TE) and culture supernatants (SN) were analysed by immunoblotting using antibodies directed against the c-Myc epitope, the translocon protein HrpF and the IM protein HrcJ, respectively. Experiments were performed three times with similar results. **(B)** Mutation of the TrM abolishes the *in vivo* function of HpaA. *X. euvesicatoria* strain 85*∆*hpaA* (∆*hpaA*) without expression construct (−) or containing HpaA-c-Myc or derivatives thereof as described in **(A)** was infiltrated into leaves of susceptible ECW and resistant ECW-10R pepper plants. Dashed lines indicate the infiltrated areas. Disease symptoms were photographed 8 dpi. For the better visualization of the HR, leaves were bleached in ethanol 2 dpi. For protein analysis, bacterial cell extracts were analysed by immunoblotting using a c-Myc epitope-specific antibody.

The finding that the TrM is essential for HpaA translocation prompted us to analyse its contribution to HpaA secretion. For this, we introduced expression constructs encoding HpaA-c-Myc and HpaA_TrMmut_-c-Myc into strain 85*∆*hpaA.* For the analysis of *in vitro* *T3S*, bacteria were incubated under *T3S*-permissive conditions, and total cell extracts and culture supernatants were analysed by immunoblotting. Figure 4A shows that HpaA-c-Myc and HpaA_TrMmut_-c-Myc were both detected in the culture supernatant, suggesting that the TrM mutation did not interfere with HpaA secretion. As expected, HpaA_TrMmut_-c-Myc restored secretion of the translocon protein HrpF in strain 85*∆*hpaA* (Figure 4A). This is in agreement with our previous observation that HpaA derivatives, which contain the C-terminal HpaB-binding site, promote secretion of translocon proteins (Lorenz et al., 2008a).

Interestingly, when bacteria were infiltrated into leaves of susceptible and resistant pepper plants, HpaA-c-Myc but not HpaA_TrMmut_-c-Myc restored pathogenicity of strain 85*∆*hpaA*, suggesting that the TrM mutation abolished the *in vivo* function of HpaA (Figure 4B). The lack of function was presumably not caused by a dominant-negative effect of HpaA_TrMmut_-c-Myc because ectopic expression of HpaA derivatives in strain 85* did not significantly interfere with pathogenicity and HR induction in susceptible and resistant plants, respectively (Figure 4B). When analysed in strain 85–10, however, both HpaA-c-Myc and HpaA_TrMmut_-c-Myc led to reduced plant reactions after infection of susceptible and resistant pepper plants (Supplementary Figure S2). We assume that enhanced levels of HpaA bind to and thus inhibit the function of HpaB. In agreement with this hypothesis, ectopic expression of HpaA deleted in the C-terminal HpaB-binding site did not significantly interfere with pathogenicity of strain 85–10 (Supplementary Figure S2). The observed dominant-negative effect of *hpaA-c-myc* and *hpaA_TrMmut_-c-myc* expression constructs likely did not interfere with protein function because ectopic expression of *hpaA-c-myc* in strain 85*∆*hpaA* restored the wild-type phenotype (Figure 4B). In summary, the results of our *in vitro* and *in vivo* experiments suggest that the TrM is dispensable for the *in vitro* secretion of HpaA but essential for the translocation and *in planta* activity of HpaA.

### Recognition of the translocation signal of HpaA depends on the HpaC-mediated *T3S* substrate specificity switch

**Figure 5:**
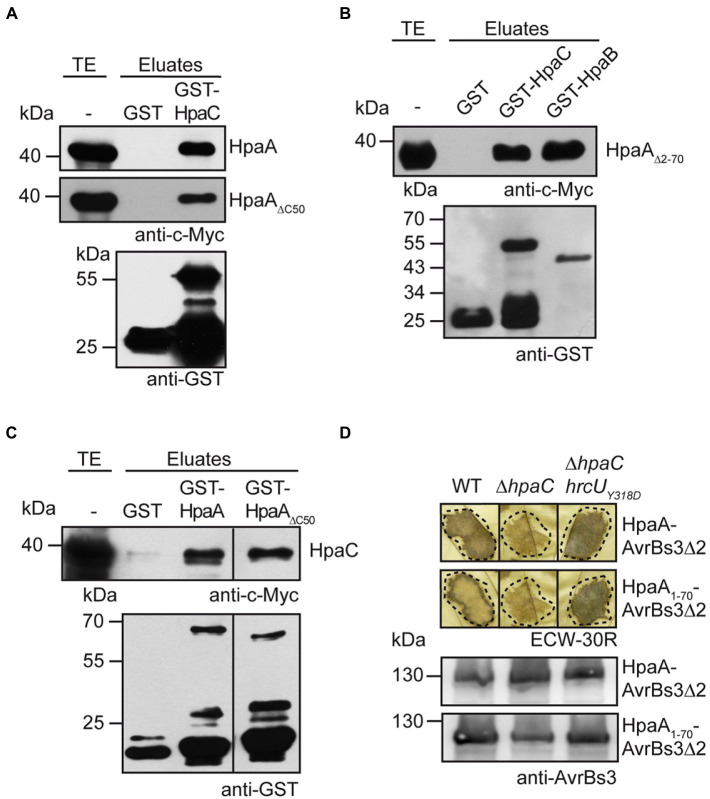
HpaA interacts with the T3S4 protein HpaC and depends on the predicted HpaC-mediated conformational change in HrcU_C_ for efficient translocation. **(A)** HpaA interacts with HpaC independently of the C-terminal HpaB-binding site. GST and GST-HpaC were immobilized on glutathione sepharose and incubated with bacterial lysates containing HpaA-c-Myc or HpaA_∆C50_-c-Myc as indicated. Total cell extracts (TE) and eluted proteins (eluates) were analysed by immunoblotting using c-Myc epitope- or GST-specific antibodies. **(B)** The N-terminal 70 amino acids of HpaA are dispensable for the interaction with HpaC. GST, GST-HpaC and GST-HpaB were immobilized on glutathione sepharose and incubated with a bacterial lysate containing HpaA_∆2-70_-c-Myc. TE and eluates were analysed as described in **(A)**. **(C)** The C-terminal HpaB-binding site in HpaA is dispensable for the interaction with HpaC. GST, GST-HpaA and GST-HpaA_∆C50_ were immobilized on glutathione sepharose and incubated with a bacterial lysate containing HpaC-c-Myc. TE and eluates were analysed as described in **(A)**. **(D)** Efficient translocation of HpaA depends on HpaC. *X. euvesicatoria* strains 85* (WT), 85*∆*hpaC* (∆*hpaC*) and 85*∆*hpaChrcU_Y318D_* (∆*hpaChrcU_Y318D_*) containing HpaA-AvrBs3∆2 or HpaA_1-70_-AvrBs3∆2 as indicated were infiltrated into leaves of AvrBs3-responsive ECW-30R pepper plants. For the better visualization of the HR, leaves were bleached in ethanol 2 dpi. Dashed lines indicate the infiltrated areas. For protein analysis, bacterial cell extracts were analysed by immunoblotting, using an AvrBs3-specific antibody.

Given the predicted function of HpaA as regulator of HpaB, we assumed that the translocation of HpaA occurs after the assembly of the *T3S* system. In *X. euvesicatoria*, secretion of translocon and T3E proteins is activated by the T3S4 protein HpaC which binds to and likely induces a conformational change in the cytoplasmic domain of the IM protein HrcU (Lorenz et al., 2008b; Lorenz and Büttner, 2011). HpaC also interacts with *T3S* substrates and the *T3S* chaperone HpaB, suggesting that it is involved in substrate recognition (Büttner et al., 2006). To investigate a possible interaction of HpaC with HpaA, we performed *in vitro* GST pull-down assays. For this, GST and a GST-HpaC fusion protein were immobilized on glutathione sepharose and incubated with an *E. coli* lysate containing HpaA-c-Myc. When eluted proteins were analysed by immunoblotting, HpaA-c-Myc was detected in the eluate of GST-HpaC but not of GST alone (Figure 5A). Similar findings were obtained for HpaA derivatives lacking the C-terminal 50 amino acids or amino acids 2–70, suggesting that the N-and C-terminal regions of HpaA are dispensable for HpaC binding (Figures 5A,B). As expected, HpaA_∆2-70_-c-Myc interacted with GST-HpaB (Figure 5B). When HpaA derivatives were analysed as GST fusions, HpaC-c-Myc coeluted with GST-HpaA and GST-HpaA_∆C50_ but not with GST (Figure 5C). We conclude from these experiments that the HpaB-binding site, which is located in the C-terminal 50 amino acids of HpaA (Lorenz et al., 2008a), is dispensable for HpaC binding, suggesting that HpaA, HpaB and HpaC can form a common protein complex.

To investigate the contribution of HpaC to HpaA translocation, we performed *in vivo* translocation assays. Translocation of HpaA-AvrBs3∆2 and HpaA_1-70_-AvrBs3∆2 was significantly reduced in strain 85*∆*hpaC* when compared with strain 85*, suggesting that the N-terminal translocation signal targets HpaA for HpaC-dependent translocation (Figure 5D). We also analysed translocation of HpaA-AvrBs3∆2 fusions in strain 85*∆*hpaChrcU_Y318D_* which contains a genomic point mutation (Y318D amino acid exchange) in the C-terminal region of HrcU (HrcU_C_). The Y318D mutation in HrcU_C_ promotes the secretion of middle and late substrates in the absence of HpaC, presumably by mimicking a predicted HpaC-induced conformational change (Lorenz and Büttner, 2011). Infection experiments showed that translocation of HpaA-AvrBs3∆2 and HpaA_1-70_-AvrBs3∆2 was restored in strain 85*∆*hpaChrcU_Y318D_*, suggesting that the HpaC-dependent translocation of HpaA depends on a certain conformation of the cytoplasmic domain of HrcU rather than on the direct interaction of HpaA with HpaC (Figure 5D). Our data hence indicate that the predicted HpaC-induced conformational change in HrcU_C_ restores recognition of the N-terminal translocation signal of HpaA in a *hpaC* deletion mutant.

### HpaA interacts with components of the predicted sorting platform and the export apparatus

**Figure 6:**
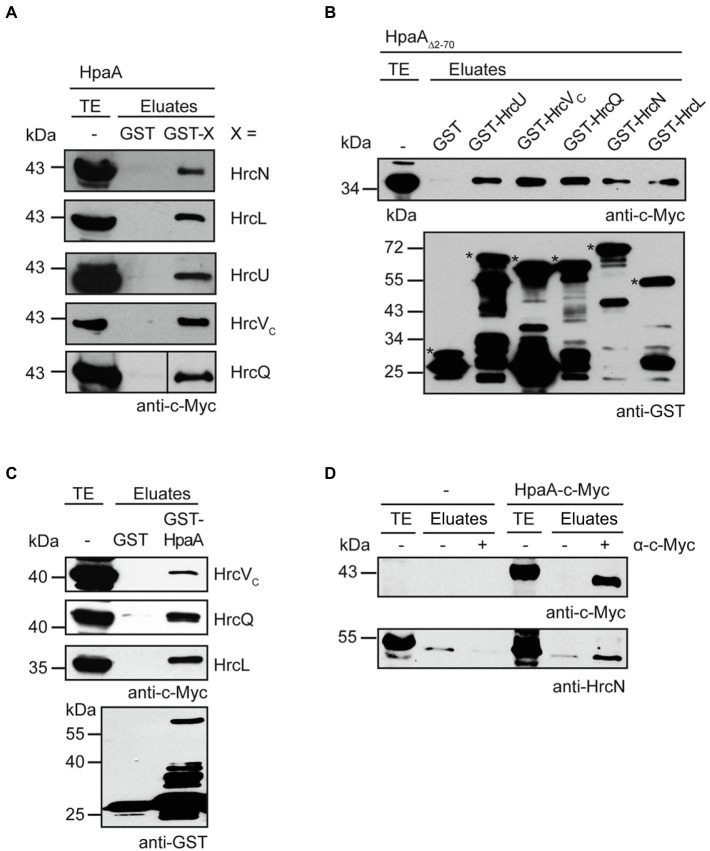
HpaA interacts with cytoplasmic components of the *T3S* system. **(A)** *In vitro* interaction studies with HpaA and cytoplasmic components of the *T3S* system. GST and GST fusions of HrcN, HrcL, HrcU, HrcV_C_ and HrcQ were immobilized on glutathione sepharose and incubated with a bacterial lysate containing HpaA-c-Myc. Total cell extracts (TE) and eluted proteins (eluates) were analysed by immunoblotting using a c-Myc epitope-specific antibody. GST and GST fusions were stably synthesized as is shown in Supplementary Figure S3. **(B)** The N-terminal region of HpaA is dispensable for the *in vitro* interaction with cytoplasmic components of the *T3S* system. GST and GST fusions as described in **(A)** were immobilized on glutathione sepharose and incubated with a bacterial lysate containing HpaA_∆2-70_-c-Myc. TE and eluates were analysed by immunoblotting using c-Myc epitope-and GST-specific antibodies. Asterisks indicate the signals corresponding to GST and GST fusion proteins. Additional signals represent degradation products. **(C)** GST-HpaA interacts with HrcV_C_, HrcQ and HrcL. GST and GST-HpaA were immobilized on glutathione sepharose and incubated with bacterial lysates containing HrcV_C_-c-Myc, HrcQ-c-Myc and HrcL-c-Myc as indicated. TE and eluates were analysed as described in **(B)**. **(D)** *In vivo* interaction studies with HpaA and HrcN. Cell lysates of *X. euvesicatoria* strain 85* without expression construct (−) or encoding HpaA-c-Myc as indicated were incubated in the absence (−) or presence (+) of a c-Myc epitope-specific antibody. TE and precipitated proteins (eluates) were analysed by immunoblotting using c-Myc epitope-and HrcN-specific antibodies. The experiment was performed three times with similar results.

The importance of the TrM for HpaA translocation suggests a possible contribution of this motif to the docking of HpaA to the *T3S* system. Potential *T3S* substrate docking sites include the ATPase HrcN, the cytoplasmic domains of the export apparatus components HrcU and HrcV as well as components of the postulated sorting platform such as HrcQ and HrcL (Lorenz and Büttner, 2009, 2011; Lorenz et al., 2012; Hartmann and Büttner, 2013). To investigate a possible docking of HpaA to components of the sorting platform and the export apparatus, we performed *in vitro* GST pull-down assays. For this, GST fusions of the ATPase HrcN, the predicted sorting platform components HrcQ and HrcL as well as the export apparatus components HrcU and the cytoplasmic domain of HrcV were immobilized on glutathione sepharose and incubated with bacterial lysates containing HpaA-c-Myc. Figure 6A shows that HpaA-c-Myc coeluted with all GST fusion proteins but not with GST alone. Similar findings were observed for a HpaA-c-Myc derivative deleted in amino acids 2–70, suggesting that the N-terminal region is dispensable for the interaction of HpaA with the analysed *T3S* system components (Figure 6B). In reciprocal experiments, c-Myc epitope-tagged derivatives of HrcQ, HrcL and the cytoplasmic domain of HrcV (HrcV_C_) coeluted with GST-HpaA but not with GST (Figure 6C). Due to protein instabilities, HrcU, HrcV and HrcN were not included in these experiments. The interaction of HpaA with HrcN was further analysed by *in vivo* co-immunoprecipitation experiments in *X. euvesicatoria* strain 85*. For this, strain 85* with or without an expression construct encoding HpaA-c-Myc was incubated under *T3S*-permissive conditions and HpaA-c-Myc was precipitated using a c-Myc epitope-specific antibody. Immunoblot analysis of precipitated proteins showed that HrcN specifically co-immunoprecipitated with HpaA-c-Myc, suggesting that both proteins are part of a common complex (Figure 6D).

### The TrM is dispensable for the interaction of HpaA with components of the *T3S* system

**Figure 7:**
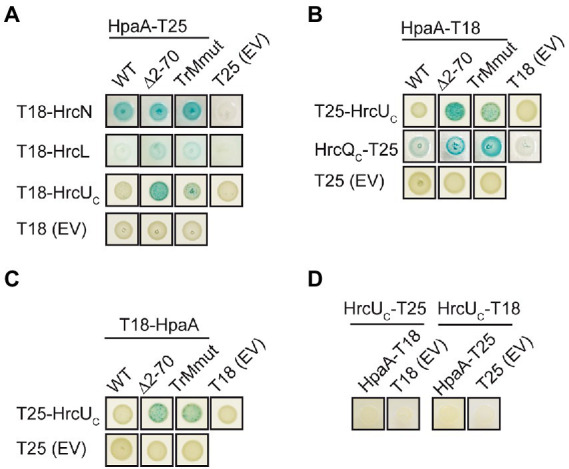
Interaction studies with HpaA using the BACTH system. **(A)** Interaction studies with HpaA, HrcN, HrcL and the cytoplasmic domain of HrcU. Expression constructs encoding T18 fusions of HrcN, HrcL and the cytoplasmic domain of HrcU (HrcU_C_) and T25 fusions of HpaA, HpaA_∆2–70_ or HpaA_TrMmut_ were transferred to *E. coli* BTH101 cells as indicated. As controls, bacteria were cotransformed with T18 or T25 fusion constructs and the corresponding empty vectors (EV) encoding T18 or T25 as indicated. Bacterial cultures were grown on indicator plates and photographs were taken after 5 days. One representative colony per interaction is shown. Cotransformations were performed three times. At least three different transformants for every combination were analysed in every experiment with similar results. All proteins were stably synthesized as is shown in Supplementary Figure S4. **(B)** Mutations in the N-terminal region of HpaA promote the interaction with HrcU_C_ and HrcQ_C_. Interactions between T18 and T25 fusions containing HpaA, HrcU_C_ and HrcQ_C_ as indicated were analysed in BTH101 cells. Bacterial cultures were grown on indicator plates and the results of the interaction assays were documented as described in **(A)**. **(C)** An N-terminal HpaA deletion derivative interacts with HrcU_C_. BTH101 cells were cotransformed with expression constructs encoding T25, T25-HrcU_C_, T18 and T18 fusions of HpaA, HpaA_∆2–70_ or HpaA_TrMmut_ as indicated. Bacterial cultures were grown on indicator plates and the results of the interaction assays were documented as described in **(A)**. **(D)** Interaction studies with HpaA and HrcU_C_. BTH101 cells were cotransformed with expression constructs encoding T25, T18 or corresponding fusions containing HrcU_C_ and HpaA as indicated. Bacterial cultures were grown on indicator plates and the results of the interaction assays were documented as described in **(A)**.

In addition to GST pull-down assays and co-immuno- precipitation experiments, we performed bacterial two hybrid-based interaction studies to confirm the interaction of HpaA with *T3S* system components and to further investigate the contribution of the TrM to the identified interactions. For this, we used the BACTH system which depends on the association of two subdomains (T18 and T25) of the catalytic domain of the adenylate cyclase (Cya) for the production of cAMP. The interaction of T18 and T25 fusion proteins leads to the reconstitution of Cya, and the subsequent cAMP production activates the expression of the *lac* operon in *E. coli* strains lacking the native *cya* gene (Karimova et al., 1998; Battesti and Bouveret, 2012). For BACTH assays, we generated expression constructs encoding HpaA and components of the *T3S* system as fusion partners of the T18 or the T25 domain. HrcN, HrcL and HrcU_C_ were analysed as C-terminal fusion partners of the T18 domain in the *E. coli* reporter strain BTH101 for interaction with HpaA-T25. When bacteria were grown on indicator plates, we observed an interaction of HpaA-T25 with T18-HrcN and a weak interaction of HpaA-T25 with T18-HrcL. In contrast, no interaction between HpaA-T25 and T18-HrcU_C_ was detected (Figure 7A). Vice versa, T25-HrcU_C_ did not detectably interact with HpaA-T18 or T18-HpaA (Figures 7B,C). Similar findings were observed for HrcU_C_ derivatives with C-terminal T18 or T25 fusion partners, suggesting that the C-terminal domain of HrcU does not interact with HpaA in BACTH assays (Figure 7D). Notably, we previously reported that HrcU_C_ interacts *in vitro* with the early *T3S* substrate HrpB2 but not with the putative translocon protein XopA or the effector protein XopC, suggesting that HrcU_C_ is not a general *T3S* substrate docking site (Lorenz and Büttner, 2011). To analyse the interaction between HpaA and HrcQ, we used the alternative translation product HrcQ_C_ which corresponds to the C-terminal domain of HrcQ (Otten et al., 2021). We observed an interaction between HrcQ_C_-T25 and HpaA-T18, suggesting that HpaA interacts with the C-terminal domain of HrcQ (Figure 7B).

We also performed BACTH assays with HpaA derivatives deleted in amino acids 2–70 or mutated in the TrM. Both derivatives interacted with HrcL and HrcN, suggesting that the N-terminal region of HpaA is dispensable for the binding of HpaA to the ATPase and HrcL (Figure 7A). Unexpectedly, deletion of the N-terminal region or mutation of the TrM enhanced the interaction of HpaA with HrcQ_C_ and HrcU_C_ whereas no interactions with T18 and T25 domains were detected (Figure 7A–C). This suggests that the N-terminal region including the TrM interferes with the efficient binding of HpaA to HrcQ_C_ and HrcU_C_. To our knowledge, a similar negative influence of a translocation signal on the interaction between a *T3S* substrate and components of the *T3S* system has not yet been described. Taken together, our data suggest that the TrM promotes the binding specificity of HpaA and might thus prevent a premature interaction of HpaA with components of the sorting platform.

## Discussion

**Figure 8:**
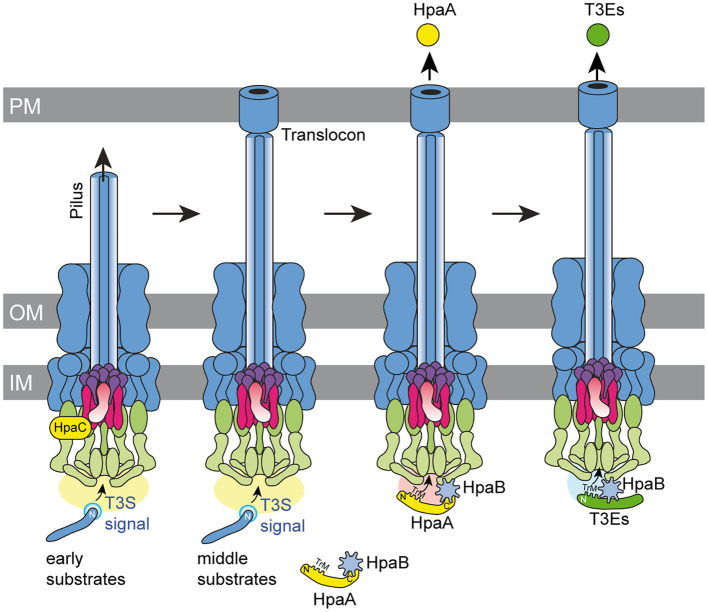
Model of the interplay between HpaA and HpaB during *T3S* in *X. euvesicatoria*. The *T3S* system assembles in both bacterial membranes and contains ring structures in the outer membrane (OM) and inner membrane (IM). The IM rings surround the export apparatus (shown in purple) and are connected to the cytoplasmic sorting platform (shown in green). The assembly and activation of the *T3S* system leads to the secretion of early substrates (i.e., the predicted inner rod protein HrpB2) and allows formation of the extracellular pilus. A switch in *T3S* substrate specificity induced by the T3S4 protein HpaC presumably involves a conformational change in the cytoplasmic domain of the export apparatus component HrcU (shown in pink) and activates the secretion of middle substrates such as components of the translocon in the plant plasma membrane (PM). The *T3S* chaperone HpaB is inactivated during assembly of the pilus and the translocon by the interaction with HpaA. A second predicted switch in *T3S* substrate specificity activates translocation of HpaA, which requires the N-terminal TrM for efficient export. Translocation of HpaA depends on HpaB, which binds to components of the *T3S* system and allows the specific recognition of the TrM, independently of its interaction with the C-terminal region of HpaA. Translocation of HpaA liberates HpaB and thus allows the translocation of T3Es, which also depend on the presence of a TrM for efficient recognition by the *T3S* system.

In the present study, we analysed *T3S* and translocation of HpaA, which is a regulator of the *T3S* chaperone HpaB and contributes to the activity of the *T3S* system (Lorenz et al., 2008a). Binding of HpaA presumably prevents an inhibitory effect of HpaB on *T3S* during the assembly of the *T3S* system and thus promotes the secretion of pilus and translocon proteins (Büttner et al., 2004; Lorenz et al., 2008a). According to this model, secretion and translocation of HpaA liberates HpaB and activates the HpaB-mediated translocation of T3Es (Lorenz et al., 2008a; Figure 8). Given the essential role of HpaA as regulator of HpaB, HpaA is likely the first protein to be translocated into the plant cell after assembly of the translocon in the plant plasma membrane. The *in planta* activity of HpaA remains to be investigated. As reported previously, HpaA contains an N-and C-terminal NLS and localizes to the plant cell nucleus when transiently expressed in *Nicotiana benthamiana* (Lorenz et al., 2008a). The contribution of HpaA to *T3S*, however, is likely attributed to its function as a regulator of HpaB in the bacterial cytosol because nuclear exclusion of HpaA did not interfere with its virulence function (Lorenz et al., 2008a).

The regulatory function of HpaA is reminiscent of the activity of the gatekeeper protein YopN (*Yersinia* outer protein N) from *Yersinia* spp., which inhibits T3E delivery when present in the cytoplasm (Iriarte et al., 1998; Amer et al., 2016). Similarly to HpaA, YopN is translocated, yet, the function of YopN in host cells remains to be determined. In the bacterial cytoplasm, YopN acts in complex with the regulator TyeA and the SycN/YscB chaperones (Cheng et al., 2001). While binding of SycN/YscB chaperones to the N-terminal region of YopN is required for its entry into the *T3S* system, binding of TyeA to the C-terminal region of YopN inhibits its transport across the bacterial membranes (Cheng et al., 2001). Host cell contact leads to the dissociation of the YopN-TyeA complex and activates secretion of YopN (Cheng and Schneewind, 2000; Cheng et al., 2001; Amer et al., 2016). Interestingly, an additional secreted control protein, YscX, was identified in *Yersinia pseudotuberculosis* and likely inhibits secretion of inner rod, needle and effector proteins when present in the cytoplasm (Gurung et al., 2022). The striking similarities between *T3S* control mechanisms involving YopN and YscX in *Yersinia* spp. and HpaA in *X. euvesicatoria* suggest that animal-and plant-pathogenic bacteria use common strategies to activate T3E delivery after assembly of the *T3S* system.

In case of HpaA, we have not yet identified a negative regulator which prevents secretion of HpaA during the formation of the *T3S* system. We assume that delivery of HpaA is activated by a yet unknown signal after assembly of the pilus and the translocon (Figure 8). In agreement with this hypothesis, we show that translocation of HpaA depends on the HpaC-mediated *T3S* substrate specificity switch which promotes secretion of translocon and effector proteins after pilus formation (Lorenz et al., 2008b). The results of our protein–protein interaction studies suggest that HpaC interacts with HpaA, yet, the importance of this interaction remains to be investigated. It is possible that the interaction of HpaA with HpaC controls the HpaC-mediated *T3S* substrate specificity switch. Notably, however, the contribution of HpaC to HpaA transport was independent of a direct protein–protein interaction and likely caused by the conformational change in HrcU_C_ which is induced by HpaC (Lorenz et al., 2008b; Lorenz and Büttner, 2011). We assume that the HpaC-mediated conformational change in HrcU_C_ first promotes secretion of translocon proteins, which are essential for translocon formation and thus T3E delivery. HpaA translocation might be activated by an additional yet unknown signal after translocon formation. It remains to be investigated whether the membrane insertion of the translocon during the natural infection or a yet unknown trigger under *in vitro* conditions generates a signal that is transmitted to the base of the *T3S* system and activates HpaA export. Interestingly, in animal-pathogenic bacteria, host cell contact likely leads to the transmission of a signal through the extracellular needle to components of the secretion apparatus in the bacterial membranes and thus activates T3E delivery (Martinez-Argudo and Blocker, 2010; El Hajjami et al., 2018; Guo et al., 2019).

We previously reported that delivery of HpaA depends on a translocation signal which is located in the N-terminal 70 amino acids (Lorenz et al., 2008a). Here, translocation studies with HpaA-reporter fusions showed that the N-terminal TrM is essential for HpaA translocation. The TrM is not highly conserved in different T3Es but contains a certain amino acid composition enriched in arginine and proline residues (Escolar et al., 2001; Prochaska et al., 2018). In the T3E XopB from *X. euvesicatoria*, the TrM was described as membrane targeting motif required for the localization of XopB at the bacterial cell membranes (Prochaska et al., 2018). The contribution of the TrM to HpaB-dependent secretion and translocation of XopB was, therefore, proposed to result from a HpaB-dependent release of XopB from the membranes. According to this model, mutation of the TrM interferes with the membrane localization of XopB and thus facilitates its secretion and translocation in the absence of HpaB (Prochaska et al., 2018). In HpaA, however, the TrM does not confer membrane localization as was revealed by the analysis of a HpaA-sfGFP fusion by fluorescence microscopy. Furthermore, as reported previously, secretion and translocation of HpaA is independent of the presence of the HpaB-binding site, suggesting that the interaction with HpaB is dispensable for efficient HpaA delivery (Lorenz et al., 2008a). This finding indicates that HpaB is not required to target HpaA to the *T3S* system.

Interestingly, our data revealed that the TrM is essential for translocation but not for *in vitro* *T3S* of HpaA, suggesting that HpaA can be recognized by the *T3S* system in the absence of the TrM. However, we assume that TrM-independent secretion of HpaA into the extracellular milieu does not occur during the natural infection. Given that pilus and translocon likely form a continuous transport channel between bacterium and plant cell, the completed assembly of the *T3S* system would leave HpaA with no other option than to be translocated. In agreement with this hypothesis, the mutation of the TrM abolished the delivery of HpaA under natural conditions. The lack of translocation results in a loss of the *in vivo* function of HpaA because cytoplasmic HpaA presumably remains attached to HpaB and thus inhibits HpaB-mediated T3E delivery. Notably, mutation of the TrM enhanced translocation of HpaA in *hpaB* mutants, suggesting that the essential contribution of the TrM to HpaA translocation depends on the presence of HpaB. This observation is reminiscent of the previous finding that the N-terminal regions of T3Es, which lack the TrM, target the AvrBs3∆2 reporter for translocation in *hpaB* mutants but not in the wild-type strain (see Table 1). The signals, which allow translocation in the absence of HpaB, were previously referred to as minimal translocation signals because they are not sufficient to promote translocation in the wild-type strain (Büttner et al., 2004; Hartmann and Büttner, 2013; Scheibner et al., 2017, 2018). We speculate that HpaB generates a docking site for the TrM after assembly of the *T3S* system and thus restricts translocation to proteins containing a TrM.

HpaB was previously shown to interact with cytoplasmic components of the *T3S* system such as components of the predicted sorting platform and the export apparatus, and might thus promote the interaction of T3Es with the *T3S* system (Lorenz and Büttner, 2009; Lorenz et al., 2012; Drehkopf et al., 2020). It is, however, still unknown how *T3S* substrates are recognized. One potential substrate docking site is HrcU_C_ which interacts with the N-terminal region of the putative inner rod protein HrpB2 (Lorenz and Büttner, 2011). Notably, however, HrcU_C_ does not interact with T3Es or the putative translocon protein XopA when analysed *in vitro*, suggesting that it is not a general *T3S* substrate docking site (Lorenz and Büttner, 2011). In the present study, we identified HrcV_C_, HrcQ_C_, HrcN and HrcL as potential docking sites for HpaA using *in vitro* and *in vivo* approaches. Furthermore, we show that HpaA and the ATPase HrcN are part of a common protein complex, suggesting that HpaA interacts with various components of the *T3S* system. Unexpectedly, the interaction of HpaA with HrcQ_C_ and HrcU_C_ appeared to be enhanced upon mutation of the TrM. It is, therefore, possible that the TrM—rather than being essential for the docking of HpaA to components of the *T3S* system—prevents an unspecific premature interaction of HpaA with components of the *T3S* system. A similar contribution of the TrM to the interaction of other T3Es with *T3S* system components remains to be investigated. Our finding sheds new light on the contribution of export signals to *T3S*. In conclusion, we propose that the TrM targets T3Es for translocation by promoting their specific interaction with *T3S* system components in the presence of HpaB and possibly preventing a premature unspecific docking to the sorting platform. Notably, a negative influence of the N-terminal region of HpaA was not observed for the interaction of HpaA with HrcN, the ATPase of the *T3S* system. HrcN presumably forms a hexameric channel through which *T3S* substrates including HpaA are transported (Lorenz and Büttner, 2009; Büttner, 2012). A negative influence of the N-terminal region of HpaA on the interaction with HrcN would, therefore, generally interfere with HpaA export.

Taken together, our data suggest that the translocation of HpaA is activated after assembly of the *T3S* system and depends on the TrM and HpaB. The precise timing of HpaA export is presumably essential for efficient T3E translocation and thus for pathogenicity of *X. euvesicatoria.* We propose that the interaction of HpaB with components of the *T3S* system generates a docking site which restricts the transit through the secretion channel to *T3S* substrates with a TrM after assembly of the *T3S* system (Figure 8). The mechanisms which activate HpaA export and control HpaB-dependent recognition of HpaA by the *T3S* system remain a challenging subject for future studies. In depth analysis of protein–protein interactions between HpaA, *T3S* system components and HpaB will help to unravel the molecular mechanisms underlying HpaA translocation and thus HpaB-dependent effector protein delivery.

## Data availability statement

The raw data supporting the conclusions of this article will be made available by the authors, without undue reservation.

## Author contributions

SD, CO and DB designed and performed the experiments and analysed the data. DB wrote the manuscript with contributions from SD and CO. All authors contributed to the article and approved the submitted version.

## Funding

This study was supported by grants from the Deutsche Forschungsgemeinschaft (BU2145/9-2 and BU2145/10-1) to DB.

## Conflict of interest

The authors declare that the research was conducted in the absence of any commercial or financial relationships that could be construed as a potential conflict of interest.

## Publisher’s note

All claims expressed in this article are solely those of the authors and do not necessarily represent those of their affiliated organizations, or those of the publisher, the editors and the reviewers. Any product that may be evaluated in this article, or claim that may be made by its manufacturer, is not guaranteed or endorsed by the publisher.

## Supplementary materials

The Supplementary materials for this article can be found online at: https://www.frontiersin.org/articles/10.3389/fpls. 2022.955776/full#supplementary-material
