# Supplementary material for: Recognition of a translocation motif in the regulator HpaA from Xanthomonas euvesicatoria is controlled by the type III secretion chaperone HpaB
Source: Front Plant Sci. 2022 Jul 28;13:955776. doi: 10.3389/fpls.2022.955776 (PMC9366055; doi:10.3389/fpls.2022.955776)
Supplement: Supplementary file 1 [file Data_Sheet_1.pdf]

Supplementary Material

Supplementary Figures and Tables

Figure S1: Immunological detection of HpaA-sfGFP and HrcC-mCherry.

Figure S2: Ectopic expression of *hpaA* exerts a dominant-negative effect on pathogenicity of strain 85-10.

Figure S3: Detection of GST and GST fusion proteins.

Figure S4: Immunological detection of T25- and T18-fusion proteins.

Table S1: Strains and plasmids used in this study.

Table S2: Oligos used in this study.

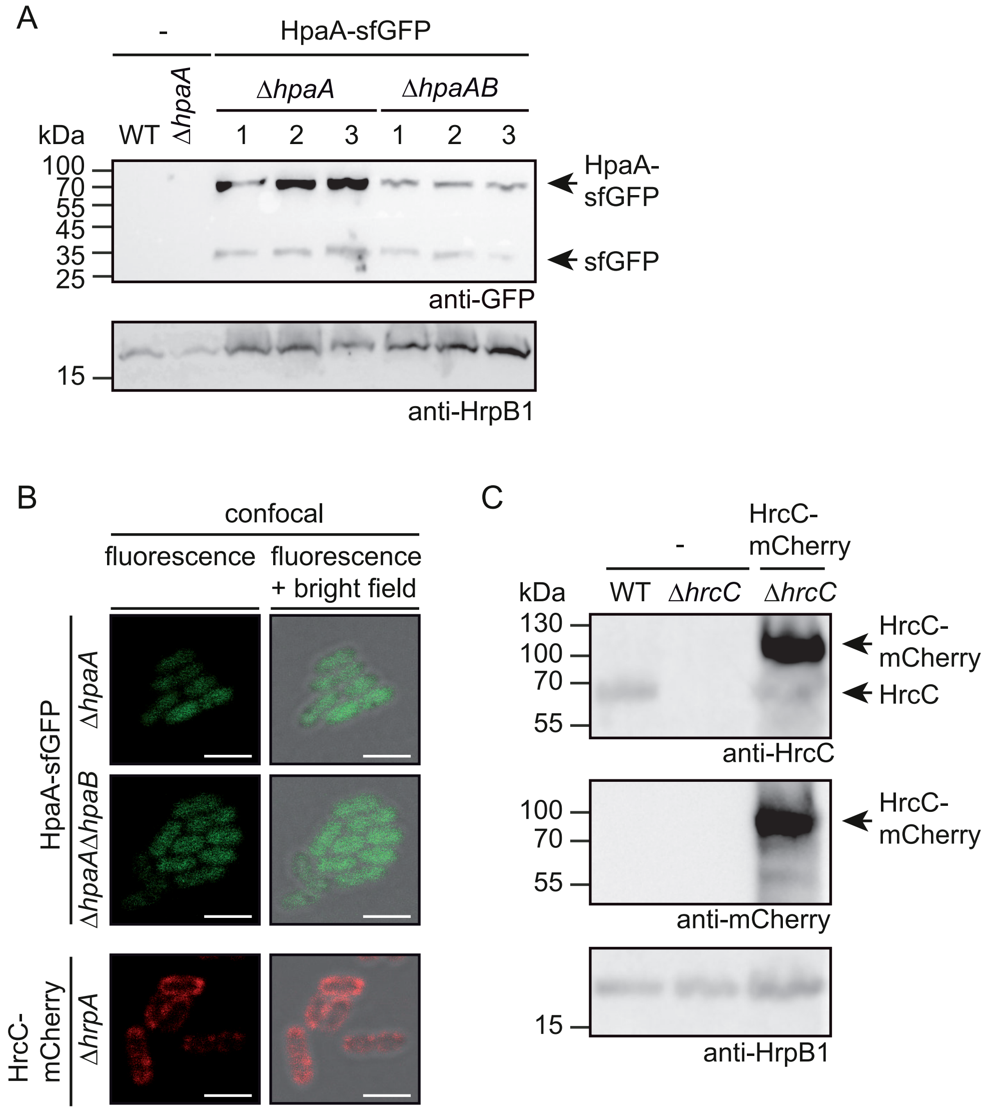

Figure S1: Immunological detection of HpaA-sfGFP and HrcC-mCherry.

(A) HpaA-sfGFP is stably synthesized. For protein analysis, three transconjugants (labeled 1, 2, and 3) of each strain were grown in minimal medium, and equal amounts of cell extracts were analysed by immunoblotting, using antibodies specific for GFP and HrpB1. The following strains were used: strain 85*Δ*hrp* containing the wild-type modular T3S gene cluster (WT) or a modular T3S gene cluster with a deletion in *hpaA* (Δ*hpaA*) and strain 85*Δ*hrp_fsHAGX* containing modular T3S gene clusters with deletions in *hpaA* or in both *hpaA* and *hpaB* (Δ*hpaAB*) and encoding HpaA-sfGFP in the flanking region of the T3S gene cluster. Signals corresponding to the full-length fusion proteins or a GFP cleavage product are indicated by arrows.

(B) Localization of HpaA-sfGFP. *X. euvesicatoria* strain 85*Δ*hrp_fsHAGX* with plasmids containing the modular T3S gene cluster, accessory and regulatory genes with deletions in *hpaA*, *hpaA* and *hpaB* or *hrcC*, and *hpaA-sfgfp* or *hrcC-mCherry* inserted into the flanking region of the modular T3S gene cluster as indicated was incubated under T3S-permissive conditions and analysed by fluorescence microscopy. One representative image for every strain is shown. The size bar corresponds to 2 µm. The pictures in the right panels result from an overlay of the signals from the fluorescent channel for GFP with the bright field images. Experiments were performed with different transconjugants for each strain three times with similar results. The results from one representative experiment are shown.

(C) Immunological detection of HrcC-mCherry. Strain 85*Δ*hrp* containing the modular T3S gene cluster with (Δ*hrcC*) or without (WT) a deletion in *hrcC* and strain 85*Δ*hrp_fsHAGX* containing the modular T3S gene cluster deleted in *hrcC* and encoding HrcC-mCherry in the flanking region of the T3S gene cluster were grown in minimal medium. Equal amounts of cell extracts of three transconjugants per strain were analysed by immunoblotting using HrcC-, mCherry- and HrpB1-specific antibodies.

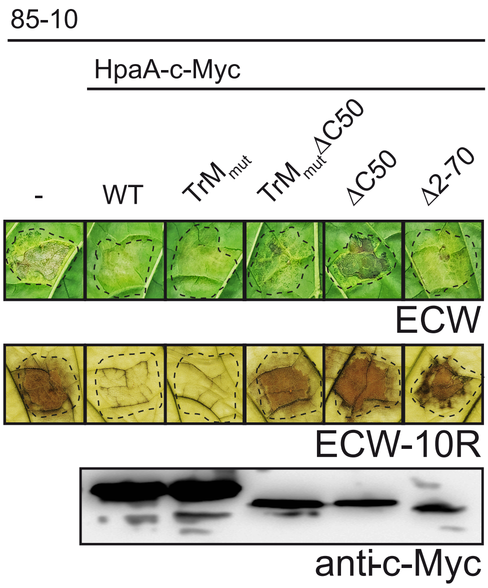

Figure S2: Ectopic expression of *hpaA* exerts a dominant-negative effect on pathogenicity of strain 85-10.

X. euvesicatoria strain 85-10 without plasmid or containing expression constructs encoding HpaA-c-Myc (WT), $HpaA_{TrMmut}$-c-Myc (TrMmut), $HpaA_{TrMmut/\Delta C50}$-c-Myc (TrMmut/ΔC50), $HpaA_{\Delta C50}$-c-Myc (ΔC50) or $HpaA_{\Delta 2\text{-}70}$-c-Myc (Δ2-70) as indicated was infiltrated into leaves of susceptible ECW and resistant ECW-10R pepper plants. Disease symptoms were photographed 8 dpi. For the better visualization of the HR, leaves were bleached in ethanol. Dashed lines indicate the infiltrated areas. For protein analysis, bacteria were grown in NYG medium and equal amounts of cell extracts were analysed by immunoblotting, using a c-Myc epitope-specific antibody.

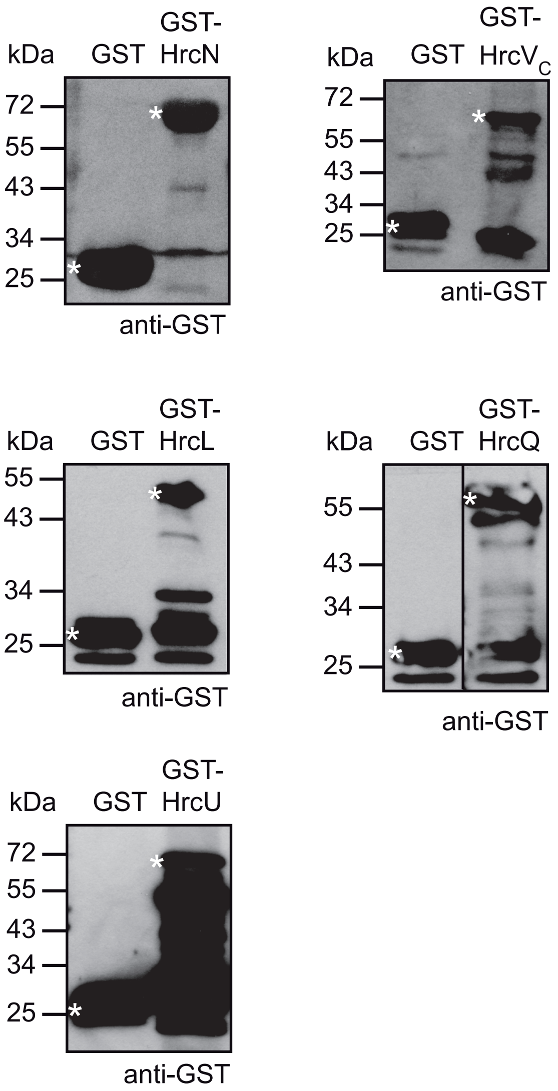

Figure S3: Detection of GST and GST fusion proteins.

Total cell extracts of bacteria containing GST or GST fusions of HrcN, HrcV$_C$, HrcL, HrcQ and HrcU as indicated were analysed by immunoblotting using a GST-specific antiserum. Asterisks indicate GST fusion proteins, additional signals represent degradation products.

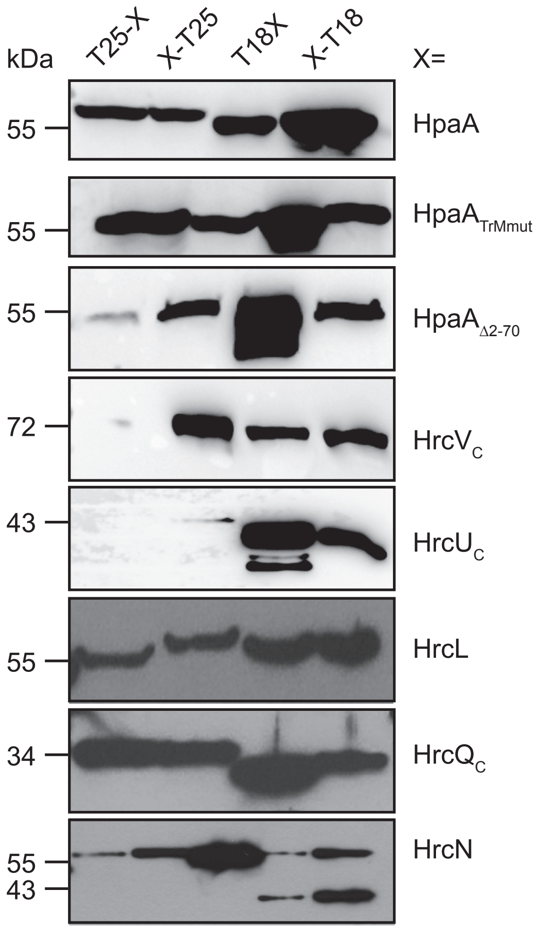

Figure S4: Immunological detection of T25- and T18-fusion proteins.

JM109 strains containing expression constructs encoding T18- and T25-fusion proteins as indicated were grown in LB medium and gene expression was induced by addition of IPTG. Total cell extracts were analysed by immunoblotting using a FLAG epitope-specific antibody.

Table S1: Strains and plasmids used in this study.

**Strain or plasmid**	**Relevant characteristics**[1]	**Reference or source**
**Strains**		
Xe		
85-10	Pepper-race 2; wild type; Rif^R	(Canteros, 1990; Kousik and Ritchie, 1998)
85*	85-10 derivative containing the *hrpG** mutation	(Wengelnik *et al.*, 1999)
85-10Δ*hpaA*	*hpaA* deletion mutant of strain 85-10	(Huguet *et al.*, 1998)
85*Δ*hpaA*	*hpaA* deletion mutant of strain 85*	(Lorenz *et al.*, 2008a)
85*Δ*hpaB*	*hpaB* deletion mutant of strain 85*	(Büttner *et al.*, 2004)
85*Δ*hpaC*	*hpaC* deletion mutant of strain 85*	(Büttner *et al.*, 2006)
85*Δ*hpaChrcU*$_{Y318D}$	*hrcU*$_{Y318D}$ mutant derivative of strain 85*Δ*hpaC*	(Lorenz and Büttner, 2011)
85*Δ*hrp*	Derivative of strain 85* with a complete deletion of the T3S gene cluster	(Hausner *et al.*, 2019)
85*Δ*hrp*_fs*HAGX*	Derivative of strain 85*Δ*hrp* with frameshift mutations after codons 26 of *hrpX*, 12 of *hrpG*, 16 of *xopA* and 7 of *hpaH*	(Hausner *et al.*, 2019)
E. coli		
OneShot®TOP10	F$^-$, *mcrAΔ(mrr-hsdRMS-mcrBC)*, *Φ80lacZΔM15*, Δ*lacX74*, *recA1*, araΔ*139*Δ*(ara-leu)7697*, *galU*, *galK*, *rpsL*, *endA1, nupG*	Invitrogen
DH5α λpir	F$^-$ *recA hsdR17*(r$_K^-$,m$_K^+$) ϕ*80dlacZ DM15 [λpir]*	(Ménard *et al.*, 1993)
BL21 (DE3)	F$^-$, *ompT, hsdSB* (r$_B^-$m$_B^-$), *gal,dcm (DE3)*	Stratagene
JM109	F$^-$, *traD36 proA$^+$B$^+$ lacI^q* Δ*(lacZ)M15*/ Δ*(lac-proAB) glnV44 e14$^-$ gyrA96 recA1 relA1 endA1 thi hsdR17*	(Yanisch-Perron *et al.*, 1985)
BTH101	F$^-$, *cya-99*, *araD139*, *galE15*, *galK16*, *rpsL1* (Str^R), *hsdR2*, *mcrA1*, *mcrB1*	(Euromedex; Battesti and Bouveret, 2012)
**Plasmids**		
pBBR1MCS-5	Broad-host-range vector; *lac* promoter; Gm^R	(Kovach *et al.*, 1995)
pBRM	Golden Gate-compatible derivative of pBBR1MCS-5 containing the *lac* promoter, a *lacZα* fragment flanked by *Bsa*I recognition sites and a 3 x c-Myc epitope-encoding sequence; Gm^R	(Szczesny *et al.*, 2010)
pBhpaA	Derivative of pBRM encoding HpaA-c-Myc	This study
pBhpaA$_{ΔC50}$	Derivative of pBRM encoding HpaA$_{ΔC50}$-c-Myc	This study
pBhpaA$_{TrMmut}$	Derivative of pBRM encoding HpaA$_{TrMmut}$-c-Myc	This study
pBhpaA$_{Δ2-70}$	Derivative of pBRM encoding HpaA$_{Δ2-70}$-c-Myc	This study
pBhrcV$_{324-645}$	Derivative of pBRM encoding HrcV$_{324-645}$-c-Myc	(Hartmann and Büttner, 2013)
pBR356	Derivative of plasmid pBBR1MCS-5 containing *avrBs3*Δ*2* downstream of the *lac* promoter and the *lacZ*α fragment, which is flanked by *BsaI* sites	(Scheibner *et al.*, 2016)
pBhpaA-356	Derivative of pBR356 encoding HpaA- AvrBs3*Δ*2	This study
pBhpaA$_{ΔC50}$-356	Derivative of pBR356 encoding HpaA$_{ΔC50}$-AvrBs3*Δ*2	This study
pBhpaA$_{TrMmut}$-356	Derivative of pBR356 encoding HpaA$_{TrMmut}$-AvrBs3*Δ*2	This study
pBhpaA$_{Δ2-70}$-356	Derivative of pBR356 encoding HpaA$_{Δ2-70}$-AvrBs3*Δ*2	This study
pBhpaA$_{1-50}$-356	Derivative of pBR356 encoding HpaA$_{1-50}$-AvrBs3*Δ*2	This study
pBhpaA$_{1-60}$-356	Derivative of pBR356 encoding HpaA$_{1-60}$-AvrBs3*Δ*2	This study

pBhpaA$_{1-70}$-356	Derivative of pBR356 encoding HpaA$_{1-70}$-AvrBs3*Δ*2	This study
pBhpaA$_{1-70/TrMmut}$-356	Derivative of pBR356 encoding HpaA$_{1-70/TrMmut}$-AvrBs3*Δ*2	This study
pGEX-2TKM	*gst* expression vector; p$_{tac}$ GST *lacI*^{q} pBR322 *ori*; Ap^{R}, derivative of pGEX-2TK with polylinker of pDSK604	(Stratagene; Escolar *et al.*, 2001)
pGhpaA	Derivative of pGEX-2TKM encoding GST-HpaA	(Lorenz *et al.*, 2008a)
pGhpaA $_{Δ2-70}$	Derivative of pGEX-2TKM encoding GST-HpaA $_{Δ2-70}$	(Lorenz *et al.*, 2008a)
pGhpaB	Derivative of pGEX-2TKM encoding GST-HpaB	(Büttner *et al.*, 2006)
pGhpaC	Derivative of pGEX-2TKM encoding GST-HpaC	(Büttner *et al.*, 2006)
pGhrcN	Derivative of pGEX-2TKM encoding GST-HrcN	(Lorenz, 2009)
pGhrcL	Derivative of pGEX-2TKM encoding GST-HrcL	(Lorenz and Büttner, 2009)
pGhrcQ	Derivative of pGEX-2TKM encoding GST-HrcQ	(Lorenz *et al.*, 2012)
pGhrcU	Derivative of pGEX-2TKM encoding GST-HrcU	(Lorenz *et al.*, 2008b)
pGhrcV$_{324-645}$	Derivative of pGEX-2TKM encoding GST-HrcV$_{324-645}$	(Hartmann and Büttner, 2013)
pDSK602	Broad-host-range vector; *lacUV5*-promoter; Spec^{R}	(Murillo *et al.*, 1994)
pDMhpaC	Derivative of pDSK602 encoding HpaC-c-Myc	(Büttner *et al.*, 2006)
pDMhrcL	Derivative of pDSK602 encoding HrcL-c-Myc	(Lorenz and Büttner, 2009)
pDMhrcQ	Derivative of pDSK602 encoding HrcQ-c-Myc	C. Lorenz and D. Büttner, unpublished
BACTH vectors		
pUT18	BACTH vector, derivative of pUC19, encodes the T18 fragment (amino acids 225 – 399) of CyaA downstream of a *lac* promoter and a multiple cloning site for classical cloning, Ap^{R}	(Euromedex; Karimova *et al.*, 2001)
pUT18C	BACTH vector, derivative of pUT18, encodes the T18 fragment (amino acids 225 – 399) of CyaA downstream of a *lac* promoter; the multiple cloning site for classical cloning is inserted at the 3' end of the T18-encoding fragment, Ap^{R}	(Euromedex; Karimova *et al.*, 2001)
pKT25	BACTH vector, derivative of low copy number plasmid pSU40, encodes the T25 fragment (first 224 amino acids) of CyaA downstream of a *lac* promoter, the multiple cloning site for classical cloning is inserted at the 3' end of the T25 encoding fragment, Km^{R}	(Euromedex; Karimova *et al.*, 2001)
pKNT25	BACTH vector, derivative of pKT25, contains the T25-encoding fragment downstream of a multiple cloning site for classical cloning and the *lac* promoter, Km^{R}	(Euromedex; Karimova *et al.*, 2001)
pUT18$_{GG}$	Golden Gate-compatible derivative of pUT18 containing *lacP-eforRed* flanked by *Bsa*I sites upstream of the *FLAG-T18* fragment; Gm^{R}	(Otten and Büttner, 2021)
pUT18C$_{GG}$	Golden Gate-compatible derivative of pUT18C containing *lacP-eforRed* flanked by *BsaI* sites downstream of the *T18-FLAG* fragment; Gm^{R}	(Otten and Büttner, 2021)
pKT25$_{GG}$	Golden Gate-compatible derivative of pKT25; contains *lacP-eforRed* flanked by *BsaI* sites downstream of the *T25-FLAG* fragment; Km^{R}	(Otten and Büttner, 2021)

pKNT25$_{GG}$	Golden Gate-compatible derivative of pKNT25, contains *lacP-eforRed* flanked by *BsaI* sites upstream of the *T25-FLAG* fragment; Km^{R}	(Otten and Büttner, 2021)
pUT18$_{GG}$hpaA	Derivative of pUT18$_{GG}$ encoding HpaA-FLAG-T18	This study
pUT18C$_{GG}$hpaA	Derivative of pUT18C$_{GG}$ encoding T18-FLAG-HpaA	This study
pKNT25$_{GG}$hpaA	Derivative of pKNT25$_{GG}$ encoding HpaA-FLAG-T25	This study
pKT25$_{GG}$hpaA	Derivative of pKT25$_{GG}$ encoding T25-FLAG-HpaA	This study
pUT18$_{GG}$hpaA$_{\Delta 2-70}$	Derivative of pUT18$_{GG}$ encoding HpaA$_{\Delta 2-70}$-FLAG-T18	This study
pUT18C$_{GG}$hpaA$_{\Delta 2-70}$	Derivative of pUT18C$_{GG}$ encoding T18-FLAG-HpaA$_{\Delta 2-70}$	This study
pKNT25$_{GG}$hpaA$_{\Delta 2-70}$	Derivative of pKNT25$_{GG}$ encoding HpaA$_{\Delta 2-70}$-FLAG-T25	This study
pKT25$_{GG}$hpaA$_{\Delta 2-70}$	Derivative of pKT25$_{GG}$ encoding T25-FLAG-HpaA$_{\Delta 2-70}$	This study
pUT18$_{GG}$hpaA$_{TrMmut}$	Derivative of pUT18$_{GG}$ encoding HpaA$_{TrMmut}$-FLAG-T18	This study
pUT18 $_{GG}$hpaA$_{TrMmut}$	Derivative of pUT18C$_{GG}$ encoding T18-FLAG-HpaA$_{TrMmut}$	This study
pKNT2 $_{GG}$hpaA$_{TrMmut}$	Derivative of pKNT25$_{GG}$ encoding HpaA$_{TrMmut}$-FLAG-T25	This study
pKT25$_{GG}$hpaA$_{TrMmut}$	Derivative of pKT25$_{GG}$ encoding T25-FLAG-HpaA$_{TrMmut}$	This study
pUT18$_{GG}$hpaB	Derivative of pUT18$_{GG}$ encoding HpaB-FLAG-T18	This study
pUT18C$_{GG}$hpaB	Derivative of pUT18C$_{GG}$ encoding T18-FLAG-HpaB	This study
pKNT25$_{GG}$hpaB	Derivative of pKNT25$_{GG}$ encoding HpaB-FLAG-T25	This study
pKT25$_{GG}$hpaB	Derivative of pKT25$_{GG}$ encoding T25-FLAG-HpaB	This study
pUT18$_{GG}$hrcL	Derivative of pUT18$_{GG}$ encoding HrcL-FLAG-T18	(Otten and Büttner, 2021)
pUT18C$_{GG}$hrcL	Derivative of pUT18C$_{GG}$ encoding T18-FLAG-HrcL	(Otten and Büttner, 2021)
pKNT25$_{GG}$hrcL	Derivative of pKNT25$_{GG}$ encoding HrcL-FLAG-T25	(Otten and Büttner, 2021)
pKT25$_{GG}$hrcL	Derivative of pKT25$_{GG}$ encoding T25-FLAG-HrcL	(Otten and Büttner, 2021)
pUT18$_{GG}$hrcQ$_{C}$	Derivative of pUT18$_{GG}$ encoding HrcQ$_{C}$-FLAG-T18	(Otten *et al.*, 2021)
pUT18C$_{GG}$hrcQ$_{C}$	Derivative of pUT18C$_{GG}$ encoding T18-FLAG-HrcQ$_{C}$	(Otten *et al.*, 2021)
pKNT25$_{GG}$hrcQ$_{C}$	Derivative of pKNT25$_{GG}$ encoding HrcQ$_{C}$-FLAG-T25	(Otten *et al.*, 2021)
pKT25$_{GG}$hrcQ$_{C}$	Derivative of pKT25$_{GG}$ encoding T25-FLAG-HrcQ$_{C}$	(Otten *et al.*, 2021)
pUT18$_{GG}$hrcU$_{C}$	Derivative of pUT18$_{GG}$ encoding HrcU$_{C}$-FLAG-T18	This study
pUT18C$_{GG}$hrcU$_{C}$	Derivative of pUT18C$_{GG}$ encoding T18-FLAG-HrcU$_{C}$	This study
pKNT25$_{GG}$hrcU$_{C}$	Derivative of pKNT25$_{GG}$ encoding HrcU$_{C}$-FLAG-T25	This study
pKT25$_{GG}$hrcU$_{C}$	Derivative of pKT25$_{GG}$ encoding T25-FLAG-HrcU$_{C}$	This study
pUT18$_{GG}$hrcN	Derivative of pUT18$_{GG}$ encoding HrcN-FLAG-T18	(Otten and Büttner, 2021)
pUT18C$_{GG}$hrcN	Derivative of pUT18C$_{GG}$ encoding T18-FLAG-HrcN	(Otten and Büttner, 2021)
pKNT25$_{GG}$hrcN	Derivative of pKNT25$_{GG}$ encoding HrcN-FLAG-T25	(Otten and Büttner, 2021)
pKT25$_{GG}$hrcN	Derivative of pKT25$_{GG}$ encoding T25-FLAG-HrcN	(Otten and Büttner, 2021)

**Constructs for modular cloning**		
Destination vectors		
pAGM9121	pUC19-derived vector, *lacZα* fragment flanked by *Bpi*I sites; Sm^R	(Addgene #51833; Weber *et al.*, 2011)
pAGM1311	pUC19-derived level -1 vector, *lacZα* fragment flanked by *Bsa*I sites; Km^R	(Addgene #47983; Weber *et al.*, 2011)
pICH41021	Derivative of pUC19 with mutated *Bsa*I site; Ap^R	Gift from S. Marillonnet
pICH41276	pUC19-derived level 0 vector, *lacZα* fragment flanked by *Bpi*I sites; external *Bsa*I sites generate GCTT / CGCT overhangs; Sm^R	(Addgene #47994; Weber *et al.*, 2011)
pICH41295	pUC19-derived level 0 vector, *lacZα* fragment flanked by *Bpi*I sites; external *Bsa*I sites generate GGAG / AATG overhangs; Sm^R	(Addgene #47997; Weber *et al.*, 2011)
pICH41308	pUC19-derived level 0 vector, *lacZα* fragment flanked by *Bpi*I sites; external *Bsa*I sites generate AATG / GCTT overhangs; Sm^R	(Addgene #47998; Weber *et al.*, 2011)
pICH41331	pUC19-derived level 0 vector, *lacZα* fragment flanked by *Bpi*I sites; external *Bsa*I sites generate GGAG / CGCT overhangs; Sm^R	(Addgene #47999; Weber *et al.*, 2011)
pICH47742	Level 1 destination vector derived from pBIN19 and pUC19, *lacZα* fragment flanked by *Bsa*I sites, for level M position 2'; Ap^R	(Addgene #48001; Weber *et al.*, 2011)
pICH47751	Level 1 destination vector derived from pBIN19 and pUC19, *lacZα* fragment flanked by *Bsa*I sites, for level M position 3; Ap^R	(Addgene #48002; Weber *et al.*, 2011)
pICH47781	Level 1 destination vector derived from pBIN19 and pUC19, *lacZα* fragment flanked by *Bsa*I sites, for level M position 6; Ap^R	(Addgene #48005; Weber *et al.*, 2011)
pICH47811	Level 1 destination vector derived from pBIN19 and pUC19, *lacZα* fragment flanked by *Bsa*I sites, for level M position 2; Ap^R	(Addgene #48008; Weber *et al.*, 2011)
pAGM8031	Level M vector derived from pBIN19 and pUC19, *lacZα* fragment flanked by *Bpi*I sites; Sm^R	(Addgene #48037; Weber *et al.*, 2011)
pAGM8079	Level M vector derived from pBIN19 and pUC19, *lacZα* fragment flanked by *Bpi*I sites; Sm^R	(Addgene #48041; Weber *et al.*, 2011)
pICH75322	Level P vector derived from pPZP200 and pUC19, *lacZα* fragment flanked by *Bsa*I sites, ColE1 and pVS1 ori, Km^R	(Addgene #48051; Weber *et al.*, 2011)
**End linker constructs**		
pICH79264	Derived from pUC19, Ap^R, level P end linker for position 3; ACTA / GGGA *Bsa*I and ACTA/- *Bpi*I fusion sites	(Addgene #48059; Weber *et al.*, 2011)
pICH50881	Derived from pUC19, Ap^R, level M end linker for position 3; ACTA/- *Bsa*I and ACTA/ GGGA *Bpi*I fusion sites	(Addgene #48045; Weber *et al.*, 2011)
pICH50900	Derived from pUC19, Ap^R, level M end linker for position 5; CAGA/ - *Bsa*I and CAGA/ GGGA *Bpi*I fusion sites	(Addgene #48047; Weber *et al.*, 2011)
**Dummy modules**		
pICH54011	Derived from pBIN19 and pUC19, 15 bp insert for level M position 1 with TGCC/ GCAA *Bpi*I fusion sites, Ap^R	(Addgene #48065; Weber *et al.*, 2011)
pICH54022	Derived from pBIN19 and pUC19, 15 bp insert for level M position 2' with GCAA/ ACTA *Bpi*I fusion sites, Ap^R	(Addgene #48066; Weber *et al.*, 2011)

pICH54066	Derived from pBIN19 and pUC19, 15 bp insert for level M position 6 with TGTG/ GAGC *Bpi*I fusion sites, Ap^R	(Addgene #48070; Weber *et al.*, 2011)
**Level -2 constructs**		
pAGB192	Level -2 construct; derivative of pAGM9121 containing *hrcL*; Sm^R	(Hausner *et al.*, 2019)
pAGB193	Level -2 construct; derivative of pAGM9121 containing *hrcN*; Sm^R	(Hausner *et al.*, 2019)
pAGB194	Level -2 construct; derivative of pAGM9121 containing *hrpB7*; Sm^R	(Hausner *et al.*, 2019)
pAGB195	Level -2 construct; derivative of pAGM9121 containing *hrcT*; Sm^R	(Hausner *et al.*, 2019)
pAGB205	Level -2 construct; derivative of pAGM9121 containing *hrcS*; Sm^R	(Hausner *et al.*, 2019)
pAGB207	Level -2 construct; derivative of pAGM9121 containing *hrpD6*; Sm^R	(Hausner *et al.*, 2019)
pAGB208	Level -2 construct; derivative of pAGM9121 containing *hrpE* and *hpaB*; Sm^R	(Hausner *et al.*, 2019)
pAGB209	Level -2 construct; derivative of pAGM9121 containing *hpaE*; Sm^R	(Hausner *et al.*, 2019)
pAGB515	Level -2 construct; derivative of pAGM9121 containing only 204 bp downstream and 44 bp upstream of *hrcC* (complete deletion of *hrcC*); Sm^R	This study
pAGB611	Level -2 construct; derivative of pICH41021 containing *hrcC* including the stop codon and lacking the start codon for generation of N-terminal fusions; Ap^R	This study
pAGB649	Level -2 construct; derivative of pAGM9121 containing *hrpE* Sm^R	This study
pAGB650	Level -2 construct; derivative of pAGM9121 containing the first 51 bp of *hpaB* with an additional nucleotide after the start codon leading to a frameshift mutation; Sm^R	This study
pAGB1302	Level -2 construct; derivative of pAGM9121 containing *hpaA* including the stop codon and lacking the start codon for generation of N-terminal fusions; Sm^R	This study
pAGB1323	Level -2 construct; derivative of pAGM9121 containing *hrcD* and *hpaA* deleted in bp 7 - 546; Sm^R	This study
**Level -1 constructs**		
pAGB197	Level -1 construct; derivative of pAGM1311 containing *hrpB1* to *hrpB4*; Km^R	(Hausner *et al.*, 2019)
pAGB210	Level -1 construct; derivative of pAGM1311 containing the *hrpC* and *hrpD* operon without *hrcS* and *hpaA*; Km^R	(Hausner *et al.*, 2019)
pAGB474	Level -1 construct; derivative of pAGM1311 containing *hrcC* including the stop codon and lacking the start codon for generation of N-terminal fusions; Km^R	This study
pAGB614	Level -1 construct; derivative of pICH41021 containing the native *hrpA* operon promoter; Ap^R	This study
pAGB676	Level -1 construct; derivative of pAGM1311 containing *hrcL*, *hrcN*, *hrpB7*, *hrcT* and *hrcC* with a deletion in *hrcC*; Km^R	This study
pAGB997	Level -1 construct; derivative of pICH41021 containing *sfgfp* for generation of C-terminal fusions; Ap^R	(Otten *et al.*, 2021)

pAGB1000	Level -1 construct; derivative of pICH41021 containing a linker (2 x AKLEGPAGL)-encoding sequence; Ap^R	(Otten *et al.*, 2021)
pAGB1048	Level -1 construct; derivative of pICH41021 containing *mCherry* for generation of C-terminal fusions; Ap^R	This study
pAGB1304	Level -1 construct; derivative of pAGM1311 containing *hpaA* including the stop codon and lacking the start codon for generation of N-terminal fusions; Km^R	This study
pAGB1324	Level -1 construct; derivative of pAGM1311 containing the *hrcS* – *hpaE* region with a deletion in *hpaA*; Km^R	This study
pAGB1359	Level -1 construct; derivative of pAGM1311 containing the *hrcS* – *hpaE* region with a deletion in *hpaA* and *hpaB*; Km^R	This study
**Level 0 constructs**		
pAGB232	Level 0 construct; derivative of pICH41276 containing a transcriptional terminator (*Xcv term2*); Sm^R	This study
pAGB249	Level 0 construct; derivative of pICH41295 containing the native *hrpD* operon promoter; Sm^R	(Hausner *et al.*, 2019)
pAGB511	Level 0 construct; derivative of pICH41295 containing the native *hrpA* operon promoter; Sm^R	This study
pAGB680	Level 0 construct; derivative of pICH41331 containing the *hrpA* and *hrpB* operons with a deletion in *hrcC*; Sm^R	This study
pAGB1059	Level 0 construct; derivative of pICH41308 containing *hrcC-2xAKLEGPAGL-mCherry*; Sm^R	This study
pAGB1325	Level 0 construct; derivative of pICH41331 containing the *hrpC*, *hrpD*, *hrpE* and *hpaB* operons operons with a deletion in *hpaA*; Sm^R	This study
pAGB1328	Level 0 construct; derivative of pICH41308 containing *hpaA-2xAKLEGPAGL-sfgfp*; Sm^R	This study
pAGB1360	Level 0 construct; derivative of pICH41331 containing the *hrpC*, *hrpD*, *hrpE* and *hpaB* operons operons with a deletion in *hpaA* and *hpaB*; Sm^R	This study
**Level 1 constructs**		
pAGB154	Level 1 construct; derivative of pICH47811 containing the *hrpA* and *hrpB* operon; Ap^R	(Hausner *et al.*, 2019)
pAGB155	Level 1 construct; derivative of pICH47751 containing the *hrpC*, *hrpD*, *hrpE* and *hpaB* operons; Ap^R	(Hausner *et al.*, 2019)
pAGB156	Level 1 construct; derivative of pICH47761 containing the *hrpF* operon; Ap^R	(Hausner *et al.*, 2019)
pAGB157	Level 1 construct; derivative of pICH47772 containing *xopA* and *hpaH*; Ap^R	(Hausner *et al.*, 2019)
pAGB160	Level 1 construct; derivative of pICH47861 containing *hrpX*; Ap^R	(Hausner *et al.*, 2019)
pAGB163	Level 1 construct; derivative of pICH47732 containing *hrpG**; Ap^R	(Hausner *et al.*, 2019)
pAGB683	Level 1 construct; derivative of pICH47811 containing the *hrpA* and *hrpB* operons deleted in *hrcC*; Ap^R	This study
pAGB1063	Level 1 construct; derivative of pICH47781 containing *hrcC-2xAKLEGPAGL-mcherry* downstream of the native *hrpA* operon promoter; Ap^R	This study

pAGB1326	Level 1 construct; derivative of pICH47751 containing the *hrpC*, *hrpD*, *hrpE* and *hpaB* operons with a deletion in *hpaA*; Ap^{R}	This study
pAGB1330	Level 1 construct; derivative of pICH47742 containing *hpaA-2xAKLEGPAGL-sfgfp* downstream of the native *hrpD* operon promoter; Ap^{R}	This study
pAGB1361	Level 1 construct; derivative of pICH47751 containing the *hrpC*, *hrpD*, *hrpE* and *hpaB* operons with a deletion in *hpaA* and *hpaB*; Ap^{R}	This study
**Level M constructs**		
pAGB168	Level M construct; derivative of pAGM8031 containing the *hrp* gene cluster; Sm^{R}	(Hausner *et al.*, 2019)
pAGB739	Level M construct; derivative of pAGM8031 containing the *hrp* gene cluster deleted in *hrcC*; Sm^{R}	This study
pAGB1067	Level M construct; derivative of pAGM8079 containing *xopA*, *hpaH*, *hrcC-2xAKLEGPAGL - mCherry*, *hrpX* and *hrpG**; Sm^{R}	This study
pAGB1327	Level M construct; derivative of pAGM8031 containing the *hrp* gene cluster deleted in *hpaA*; Sm^{R}	This study
pAGB1332	Level M construct; derivative of pAGM8079 containing *xopA*, *hpaH*, *hpaA-2xAKLEGPAGL-sfGFP*, *hrpX* and *hrpG**; Sm^{R}	This study
pAGB1362	Level M construct; derivative of pAGM8031 containing the *hrp* gene cluster deleted in *hpaA* and *hpaB*; Sm^{R}	This study
**Level P constructs**		
pAGB1075	Level P construct; derivative of pICH75322 containing the *hrp* gene cluster (with deletions in *hrcC*), *xopA*, *hpaH*, *hrcC-2xAKLEGPAGL - mCherry*, *hrpX* and *hrpG**; Km^{R}	This study
pAGB1334	Level P construct; derivative of pICH75322 containing the *hrp* gene cluster (with deletion in *hpaA*), *xopA*, *hpaH*, *hpaA-2xAKLEGPAGL-sfGFP*, *hrpX* and *hrpG**; Km^{R}	This study
pAGB1374	Level P construct; derivative of pICH75322 containing the *hrp* gene cluster (with deletions in *hpaA* and *hpaB*), *xopA*, *hpaH*, *hpaA-2xAKLEGPAGL-sfGFP*, *hrpX* and *hrpG**; Km^{R}	This study

[1] Ap, ampicillin; Gm, gentamycin; Km, kanamycin; R, resistant; Sm, spectinomycin

References

Battesti, A., and Bouveret, E. (2012). The bacterial two-hybrid system based on adenylate cyclase reconstitution in *Escherichia coli*. Methods, 58, 325-334.

Büttner, D., Gürlebeck, D., Noel, L. D., and Bonas, U. (2004). HpaB from *Xanthomonas campestris* pv. *vesicatoria* acts as an exit control protein in type III-dependent protein secretion. Mol Microbiol, 54, 755-768.

Büttner, D., Lorenz, C., Weber, E., and Bonas, U. (2006). Targeting of two effector protein classes to the type III secretion system by a HpaC- and HpaB-dependent protein complex from *Xanthomonas campestris* pv. *vesicatoria*. Mol Microbiol, 59, 513-527.

Canteros, B. I. (1990) Diversity of plasmids and plasmid-encoded phenotypic traits in *Xanthomonas campestris* pv. *vesicatoria*. PhD thesis, University of Florida

Escolar, L., Van den Ackerveken, G., Pieplow, S., Rossier, O., and Bonas, U. (2001). Type III secretion and *in planta* recognition of the *Xanthomonas* avirulence proteins AvrBs1 and AvrBsT. Mol Plant Pathol, 2, 287-296.

Hartmann, N., and Büttner, D. (2013). The inner membrane protein HrcV from *Xanthomonas* is involved in substrate docking during type III secretion. Mol Plant Microbe Interact, 26, 1176-1189.

Hausner, J., Jordan, M., Otten, C., Marillonnet, S., and Büttner, D. (2019). Modular cloning of the type III secretion gene cluster from the plant-pathogenic bacterium *Xanthomonas euvesicatoria*. ACS synthetic biology, 8, 532-547.

Huguet, E., Hahn, K., Wengelnik, K., and Bonas, U. (1998). *hpaA* mutants of *Xanthomonas campestris* pv. *vesicatoria* are affected in pathogenicity but retain the ability to induce host-specific hypersensitive reaction. Mol Microbiol, 29, 1379-1390.

Karimova, G., Ullmann, A., and Ladant, D. (2001). Protein-protein interaction between *Bacillus stearothermophilus* tyrosyl-tRNA synthetase subdomains revealed by a bacterial two-hybrid system. J Mol Microbiol Biotechnol, 3, 73-82.

Kousik, C. S., and Ritchie, D. F. (1998). Response of bell pepper cultivars to bacterial spot pathogen races that individually overcome major resistance genes. Plant Disease, 82, 181-186.

Kovach, M. E., Elzer, P. H., Hill, D. S., Robertson, M. A., Farris, M. A., Roop II, R. M. et al. (1995). Four new derivatives of the broad-host-range cloning vector pBBR1MCS, carrying different antibiotic-resistance cassettes. Gene, 166, 175-176.

Lorenz, C. (2009) Functional characterization of the conserved components HrcN und HrcU of the type III secretion system from *Xanthomonas campestris* pv. *vesicatoria*. PhD thesis. Genetics Department, Halle (Saale): Martin-Luther-University Halle-Wittenberg

Lorenz, C., and Büttner, D. (2009). Functional characterization of the type III secretion ATPase HrcN from the plant pathogen *Xanthomonas campestris* pv. vesicatoria. J Bacteriol, 191, 1414-1428.

Lorenz, C., and Büttner, D. (2011). Secretion of early and late substrates of the type III secretion system from *Xanthomonas* is controlled by HpaC and the C-terminal domain of HrcU. Mol Microbiol, 79, 447-467.

Lorenz, C., Hausner, J., and Büttner, D. (2012). HrcQ provides a docking site for early and late type III secretion substrates from *Xanthomonas*. PLoS ONE, 7, e51063.

Lorenz, C., Kirchner, O., Egler, M., Stuttmann, J., Bonas, U., and Büttner, D. (2008a). HpaA from *Xanthomonas* is a regulator of type III secretion. Mol Microbiol, 69, 344-360.

Lorenz, C., Schulz, S., Wolsch, T., Rossier, O., Bonas, U., and Büttner, D. (2008b). HpaC controls substrate specificity of the *Xanthomonas* type III secretion system. PLoS Pathog, 4, e1000094.

Ménard, R., Sansonetti, P. J., and Parsot, C. (1993). Nonpolar mutagenesis of the *ipa* genes defines IpaB, IpaC, and IpaD as effectors of *Shigella flexneri* entry into epithelial cells. J Bacteriol, 175, 5899-5906.

Murillo, J., Shen, H., Gerhold, D., Sharma, A., Cooksey, D. A., and Keen, N. T. (1994). Characterization of pPT23B, the plasmid involved in syringolide production by *Pseudomonas syringae* pv. *tomato* PT23. Plasmid, 31, 275-287.

Otten, C., and Büttner, D. (2021). HrpB4 from *Xanthomonas campestris* pv. *vesicatoria* acts similarly to SctK proteins and promotes the docking of the predicted sorting platform to the type III secretion system. Cell Microbiol, 23, e13327.

Otten, C., Seifert, T., Hausner, J., and Büttner, D. (2021). The contribution of the predicted sorting platform component HrcQ to type III secretion in *Xanthomonas campestris* pv. *vesicatoria* depends on an internal translation start site. Front Microbiol, 12, 752733.

Scheibner, F., Schulz, S., Hausner, J., Marillonnet, S., and Büttner, D. (2016). Type III-dependent translocation of HrpB2 by a non-pathogenic *hpaABC* mutant of the plant-pathogenic bacterium *Xanthomonas campestris* pv. *vesicatoria*. Appl Environ Microbiol, 82, 3331-3347.

Szczesny, R., Jordan, M., Schramm, C., Schulz, S., Cogez, V., Bonas, U. et al. (2010). Functional characterization of the Xps and Xcs type II secretion systems from the plant pathogenic bacterium *Xanthomonas campestris* pv. *vesicatoria*. New Phytol*,* 187, 983-1002.

Weber, E., Engler, C., Gruetzner, R., Werner, S., and Marillonnet, S. (2011). A modular cloning system for standardized assembly of multigene constructs. PLoS ONE*,* 6, e16765.

Wengelnik, K., Rossier, O., and Bonas, U. (1999). Mutations in the regulatory gene *hrpG* of *Xanthomonas campestris* pv. vesicatoria result in constitutive expression of all *hrp* genes. J Bacteriol*,* 181, 6828-6831.

Yanisch-Perron, C., Vieira, J., and Messing, J. (1985). Improved M13 phage cloning vectors and host strains: nucleotide sequences of the M13mp18 and pUC19 vectors. Gene*,* 33, 103-119.

Table S2: Oligos used in this study.

**Name**	**Sequence (5' → 3')** [1]
hpaA*Bsa*I-F	TTT **GGTCTCT** TATG ATCCGTCGCATCTCG
hpaA*Bsa*I-R	TTT **GGTCTCT** CACC TGGGCGAACCTCCTGAGC
hpaA(225)-R	TTT **GGTCTCT** CACC CACCGGCTCCAACTC
hpaAΔN70pBRM-F	TTT **GGTCTCT** TATG GAATTCGACGCCAACGAGC
hpaATrMmut-F	TTT **GGTCTCT** GGCA GCCGCCGGGATCCGGTC
hpaATrMmut-R	TTT **GGTCTCT** TGCC GCGGCCGGTGGAGCAG
(P)hpaADhydro-F	P-AATGCGGAAAAACCGCGCAGAC
(P)hpaADhydro-R	P-ATTGCGTAGCGCTGGCACCGGCTC
hpaA_*Bsa*_N50-R	TTT **GGTCTCT** GATC GCGCAGACGCGGTGCTC
hpaA_*Bsa*_N60-R	TTT **GGTCTCT** GATC CCCGCGGCGTCGCCTG
hpaA_*Bsa*_N70-R	TTT **GGTCTCT** GATC GAATTCATCCTCCTGGC
hpaA(70/225)-R	TTT **GGTCTCT** GCAC TTCATCCTCCTGGCCGTC
hpaA(225)-F	TTT **GGTCTCT** GTGC CAGCGCTACGCAATTTTC
pBR356hpaA-R	TTT **GGTCTCT** GATC TGGGCGAACCTCCTGAGC
hpaADN70pBR356-F	TTT **GGTCTCT** TATG GAATTCGACGCCAACGAGC
hpaB Bsa-F	TTT **GGTCTCT** TATG AGCAGCGCGCGATTCG
hpaB Bsa-R	TTT **GGTCTCT** CACC GGCGCGTACCCACAGATAG
hpaC Bsa-F	ACT **GGTCTCT** TATG CGCAAGCCGCC
hpaC Bsa-R	ACT **GGTCTCT** CACC GACAACCTCGATGCTG
hrcU 265-357-F	TTT **GGTCTCT** TATG CCGACCCATTACGCAGTG
hrcU 265-357-R	TTT **GGTCTCT** CACC GCATGGCAGGGCTCCATC
P-hrcC-Del-MoClo-F	TGATGGTTCGCGAAAAGCCATC
P-hrcC-Del-MoClo-R	GGCGTTCCCTCTGCTAGGCAAG
HpaA-Del-MoClo-F	TTT **GAAGAC** AA *CTCA* CCAT GATCTTGACCGCAACGATCTGGA GATTG
hrcD-Del-MoClo-R3	TTT **GAAGAC** AA *CTCG* TCAT TGCGCCGCTTGCTGCGGCAG
hpaB-Del-MoClo-I-F	TTT **GAAGAC** AA *CTCA* ATCG ATTTCTTGGTCGCTCGGACCAAG
hpaB-Del-MoClo-I-R	TTT **GAAGAC** AA *CTCG* CATC TGTGGTGAGACATGCAC
hpaB-Del-MoClo-II-F	TTT **GAAGAC** AA *CTCA* GATGCAGCAGCGCGCGATTCG
hpaB-Del-MoClo-II-R	TTT **GAAGAC** AA *CTCG* CTGC CAGTGCCTCGCACATCTGTC
PhrpA-MoClo-F	TTT **GAAGAC** TT *GGAG* GTGCTGGCGTTGTTGCTGCTC
PhrpA-MoClo-R	TTT **GAAGAC** TT *CATT* GGCGTTCCCTCTGCTAGGCAAGTG
hpaA-NTM-MoClo-F	TTT **GAAGAC** AA *CTCA* ACAT AATG ATCCGTCGCATCTCGCCCG
hpaA-NTM-MoClo-R	TTT **GAAGAC** AA *CTCG* ACAA TAGCTGGGCGAACCTCCTGAGCCG
hrcC-NTM-MoClo-F	TTT **GGTCTC** T *ACAT* AATG GCTCCTGCCTGTACCACCGCCCAC
hrcC-NTM-MoClo-R	TTT **GGTCTC** T *ACAA* TAGC GGGCGACACCACATGCGGGGTCA GCAG
mCherry-CTM-woL-F	TTT **GAAGAC** AA *CCTG* GTGAGCAAGGGCGAG
mCherry-CTM-woL-R	TTT **GAAGAC** AA *AAGC* TCATCA CTTGTACAGCTCGTCCATGC
termX2-euk-MoClo-F	TTT **GAAGAC** TT *GCTT* CTTCGCGCCGTCCGCATCCCGC
termX2-euk-MoClo-R	TTT **GAAGAC** TT *AGCG* CTGAGCGCGCATCATGCCAC

[1] P, phosphorylated 5' end; *Bsa*I and *Bpi*I recognition sites are shown in bold, overhangs generated by *Bsa*I and *Bpi*I restriction in italics.
